# Identification and Characterization of *LRR-RLK* Family Genes in Potato Reveal Their Involvement in Peptide Signaling of Cell Fate Decisions and Biotic/Abiotic Stress Responses

**DOI:** 10.3390/cells7090120

**Published:** 2018-08-27

**Authors:** Xiaoxu Li, Salman Ahmad, Cun Guo, Jing Yu, Songxiao Cao, Xiaoming Gao, Wei Li, Hong Li, Yongfeng Guo

**Affiliations:** 1 Key Laboratory for Tobacco Gene Resources, Tobacco Research Institute, Chinese Academy of Agricultural Sciences, Qingdao 266101, China; 82101171073@caas.cn (X.L.); safitfa@yahoo.com (S.A.); 82101172197@caas.cn (C.G.); yujing_qau@163.com (J.Y.); hanhaic@126.com (S.C.); gaoxiaoming@caas.cn (X.G.); liwei06@caas.cn (W.L.); lihongcq1993@163.com (H.L.); 2 Graduate School of Chinese Academy of Agricultural Science, Beijing 100081, China

**Keywords:** leucine-rich repeat receptor-like kinase, *Solanum tuberosum*, peptide signaling, stress response, stem cell homeostasis

## Abstract

Leucine-rich repeat receptor-like kinases (LRR-RLKs) represent the largest subfamily of receptor-like kinases (RLKs) and play important roles in regulating growth, development, and stress responses in plants. In this study, 246 *LRR-RLK* genes were identified in the potato (*Solanum tuberosum*) genome, which were further classified into 14 subfamilies. Gene structure analysis revealed that genes within the same subgroup shared similar exon/intron structures. A signature small peptide recognition motif (RxR) was found to be largely conserved within members of subfamily IX, suggesting that these members may recognize peptide signals as ligands. 26 of the 246 *StLRR-RLK* genes were found to have arisen from tandem or segmental duplication events. Expression profiling revealed that *StLRR-RLK* genes were differentially expressed in various organs/tissues, and several genes were found to be responsive to different stress treatments. Furthermore, StLRR-RLK117 was found to be able to form homodimers and heterodimers with StLRR-RLK042 and StLRR-RLK052. Notably, the overlapping expression region of *StLRR-RLK117* with *Solanum tuberosum* *WUSCHEL* (*StWUS*) suggested that the *CLV3–CLV1/BAM–WUS* feedback loop may be conserved in potato to maintain stem cell homeostasis within the shoot apical meristem.

## 1. Introduction

Unlike animals, the static build of plants handicaps their ability to escape from the hazards of environmental fluctuations. However, lacking this essential survival ability, plants have been provided with unique cell surface receptor proteins that allow the plants to perform cell-to-cell communication and to interact with their environment. In plants, these cellular receptors are known as receptor-like kinases (RLKs) which generally comprise an extracellular ligand binding domain, a transmembrane domain, and an intercellular cytoplasmic kinase domain. RLKs often serve as receptors of cell-to-cell communications by perceiving and transducing signals under various environmental conditions [1]. In *Arabidopsis*, about 610 members of RLKs have been identified previously, as a large monophyletic gene superfamily [2,3]. The structural feature of RLK proteins demonstrates a high degree of divergence in their extracellular ligand binding domain. Based on sequence analysis of the variable extracellular domain and the kinase domain, the *Arabidopsis* RLK proteins have been categorized into more than 50 different families, among which over 200 leucine-rich repeat receptor-like kinase (LRR-RLK) members represent the largest class in RLK family. The LRR-RLK ectodomains are furnished with LRR motifs, which are usually 24 amino acids long, varying in number and in arrangement. Such diversity in the LRR domain potentializes the LRR-RLK members to perceive various ligands, which also contributes towards their functional diversity [1]. Though many LRR-RLKs members had been identified, only a few of them have been characterized for their biological functions [1].

Previous studies on the biological roles of various LRR-RLK members revealed their indispensability in plant growth and developmental processes. CLAVATA 1 (CLV1), one of the LRR-RLKs in *Arabidopsis*, possesses 21 extracellular LRR domains, a transmembrane domain, and a cytoplasmic kinase domain [4]. It has been shown that the encoding gene of CLV1 is specifically expressed around the organizing center (OC) within the shoot apical meristem (SAM). It was found that CLV1 can mediate the CLV3 peptide ligand to suppress *WUSCHEL* (*WUS*) expression, whereas the transcriptional factor WUS could promote the expression of the *CLV3* gene, resulting in a negative feedback loop that is essential in maintaining stem cell homeostasis within the SAM [5,6,7]. Further, BARELY ANY MERISTEM 1 (BAM1) and BARELY ANY MERISTEM 2 (BAM2), which are both homologues of CLV1, could bind CLV3 peptides with a similar affinity, and function redundantly with CLV1 in the SAM [8,9]. In a recent study, CLAVATA3 INSENSITIVE RECEPTOR KINASES (CIKs), a group of typical LRR-RLKs, act as co-receptors of CLV1 in regulating stem cell homeostasis [10]. Besides, several LRR-RLKs have also been reported to function in peptide signaling of cell fate decisions. The *Arabidopsis* vascular meristem includes the procambium and cambium. In previous studies, *TRACHEARY ELEMENT DIFFERENTIATION FACTOR RECEPTOR/PHLOEM INTERCALATED WITH XYLEM* (*TDR/PXY*) was reported to be highly expressed in procambial cells and to encode a typical LRR-RLK, which could specifically perceive the TDIF peptide signal to promote proliferation of procambial cells, and to suppress xylem differentiation [11,12,13,14]. HAESA and HAESA-LIKE 2 (HSL2) receptors could activate the abscission and cell separation processes in *Arabidopsis* by perceiving the INFLORESCENCE DEFICIENT IN ABSCISSION (IDA) peptide signal [15]. The C-terminally encoded peptide (CEP) family was identified as a family of secreted peptides, and overexpression of the encoding genes could repress root growth [16]. The double mutant of CEPR1 and CEPR2 was insensitive to CEP1 in a root growth assay, which suggested that CEPR1 and CEPR2 function as CEP receptors [16]. The ROOT MERISTEM GROWTH FACTOR RECEPTORS (RGFRs) group of LRR-RLKs were reported to be involved in root development by sensing ROOT MERISTEM GROWTH FACTOR (RGF) peptides [17,18]. The ERECTA and ERECTA-LIKE 1 (ERL1) sense a peptide hormone named EPIDERMAL PATTERNING FACTOR 2 (EPF2) in modulating stomatal patterning [19]. In addition, phytohormone brassinosteroids bind to LRR-RLK BRASSINOSTEROID INSENSITIVE 1 (BRI1) to regulate cell elongation and cell division [20,21].

LRR-RLKs also play fundamental roles in environmental stress responses as the first line of defense by perceiving signals. In *Arabidopsis*, a number of defense related LRR-RLK members have been characterized previously. PEPR1 and its homologue PEPR2 mediate plant immunity by acting as receptors of the endogenous Pep peptides (PEP) or by sensing damage-associated molecular patterns (DAMP) [22,23]. The FLAGELLIN SENSITIVE 2 (FLS2) receptor has been reported to function in regulating defense response by sensing the bacterial flagellin monomer [24]. Another study showed that after binding flagellin, the BRASSINOSTEROID-ASSOCIATED RECEPTOR KINASE 1 (BAK1) immediately forms a sandwich structure with the C-terminus of flagellin and FLS2, which triggers downstream signaling cascades [25]. In a recent study, *Arabidopsis* LRR-RLK SIF2 (stress induced factor2) could regulate basal defense to pathogen infection by sensing a pathogen’s presence and interacting with BAK1 to activate downstream defense-related genes through the MAPK cascade [26]. Further, the RECEPTOR-LIKE PROTEIN KINASE 1 (RPK1) was reported to be involved in ABA-mediated responses to drought stress and leaf senescence in *Arabidopsis*, while the *Oryza rufipogon* homolog of RPK1, OrufRPK1 was also reported to act as a defense-related receptor [27,28,29,30].

Besides *Arabidopsis*, *LRR-RLK* genes have been identified or studied in a number of different species including *Oryza sativa*, *Solanum lycopersicum*, *Populus trichocarpa*, *Citrus clementina*, *Vitis amurensis*, and *Rosaceae species*, whereas functions of these *LRR-RLK* genes could be conserved or divergent [3,31,32,33,34,35,36]. Potato (*Solanum tuberosum*) is one of the important food crops. However, limited information is available about LRR-RLK family genes in potato. In this study, the LRR-RLK family genes of potato were studied through phylogeny, signature motif analysis, gene structure organization, chromosomal distribution, and expression profiles. The homologous counterparts between *Arabidopsis* and potato LRR-RLKs were identified to predict the potential functions of potato LRR-RLKs.

## 2. Materials and Methods

### 2.1. Identification and Phylogenetic Analysis

The potato genome annotations (PGSC, Release 3.4) were retrieved from the Sol Genomics Network (SGN, http://solgenomics.net/). All LRR-RLK full-length amino acid sequences in *Arabidopsis* were downloaded from TAIR (www.arabidopsis.org) and these sequences were used as queries to perform a BLASTP search against the potato protein database with an E-value cutoff of 0.01. These resulting sequences were then used as new queries to conduct a BLASTP search again, to avoid missing potential members. The redundant entries were removed manually, and the resulted unique sequences were then analyzed with both SMART and Pfam to ensure the presence of the LRR and RLK domains in each newly identified member. Related information, including amino acids number, molecular weights and isoelectric points, was retrieved from ProtParam (http://au.expasy.org/tools/protparam.html). Multiple sequence alignment of putative StLRR-RLKs, reported AtLRR-RLKs, and selected members from other species was performed using MAFFT, with their full-length amino acid sequences under default settings. A neighbor-joining (NJ) phylogenetic tree was generated based on the alignment result, using MEGA v6.06 with the following parameters: Poisson correction, pairwise deletion, and bootstrap values (1000 replicates). The model was advised by ProtTest 2.4. Subsequently, the tree was illustrated using FigTree 1.4.2.

### 2.2. Motif and Gene Structure Analysis

The LRR motifs were identified by MEME 4.9.1 (Multiple Expectation Maximization for Motif Elicitation, http://meme-suite.org/). Parameters were set as follows: distribution of motif occurrences, zero or one per sequence; maximum number of motifs, 40; optimum motif width, ≥22 and ≤26. The exon–intron organizations were visualized with the Gene Structure Display Server (GSDS: http://gsds.cbi.pku.edu.cn/) by comparing the coding sequences (CDSs) and genomic sequences that were obtained from the SGN database.

### 2.3. Chromosomal Localization Analysis

The positions of *StLRR-RLK* genes were analyzed and illustrated by Perl; the chromosomal localization information was retrieved from the SGN database. For nomenclature, the number was added according to the physical location on chromosomes 1–12. The tandem gene event was defined as previously described [32]: a region within 200 kb contained at least two genes which shared more than 70% identity as analyzed by BLASTP with their full-length amino acid sequences. The MCScanX program was used to identify segmental duplications as in the previous description [37,38]. These results were visualized by Circos [39].

### 2.4. Expression Profiling of StLRR-RLKs

Expression data of *StLRR-RLK* genes were retrieved from the RNA sequencing (RNA-seq) data, which was previously collected by PGSC [40]. The relative expression ratios of biotic and abiotic stress treatments were calculated relative to their controls, respectively. All expression data were normalized and visualized by R.

### 2.5. Potato Materials

The shoot cultures of potato (cultivar GN2) were maintained in our lab, and they were used for expression pattern analysis. Potato shoots were inoculated into full MS solid media by nodal cutting, and they were cultured in a growth chamber at 24 °C under continuous light. Further, these seedlings were transferred into pots filled with soil and perlite in the growth chamber, to obtain tubers. Different tissues, including shoot, shoot tip, root, root tip, young leaves, senescence leaves, and tuber were used in measuring the tissue-specific expression patterns. These samples were immediately frozen in liquid nitrogen and stored at −80 °C.

### 2.6. RNA Extraction and qRT-PCR

Total RNA from different tissues was isolated with RNAiso (TaKaRa) and first-strand complementary DNA (cDNA) synthesis was performed using 2 μg total RNA with the PrimeScript™ RT reagent Kit (TaKaRa) according to the manufacturer’s instructions. The qRT-PCR was performed on an ABI 7500 real-time PCR machine in a 20 μL reaction with SYBR (TaKaRa) 10 μL, 10 mM forward primer 0.4 μL, 10 mM reverse primer 0.4 μL, and diluted cDNA 0.2 μL. The qRT-PCR assay results were obtained from three independent replicates. The *Elongation Factor 1-α* (*EF1α*) gene was used as the internal control for normalization [41]. The relative fold differences were calculated based on the 2^−ΔΔct^ method. Primer sequences are listed in Supplementary file S1.

### 2.7. Bimolecular Fluorescence Complementation Assays

The coding sequences of *StLRR-RLK117*, *StLRR-RLK042*, and *StLRR-RLK052*, excluding their stop codons, were amplified and ligated into the entry vector pENTR by BP Clonase (Invitrogen, Carlsbad, CA, USA). The recombinant entry vectors were used to insert these sequences into pBatTL-sYFPC, mediated by LR Clonase (Invitrogen), and they were fused with the carboxy-terminal part of a yellow fluorescent protein (YFPC), resulting in the fusions to the C-termini of candidate proteins. Likewise, *StLRR-RLK117* also was inserted into pBatTL-sYFPN, and then they were fused with the amino-terminal part of the yellow fluorescent protein (YFPN), resulting in fusion to the C-termini of StLRR-RLK117. Equal cultures of StLRR-RLK117-YFPN, together with StLRR-RLK117-YFPC, StLRR-RLK042-YFPC, StLRR-RLK052-YFPC, and the negative control AtBRI1-YFPC, were injected into *Nicotiana benthamiana* leaves separately for *Agrobacterium*-mediated transient expression. Fluorescence signals of the reconstituted YFP were captured using a confocal microscope (TCS-SP8 Leica, Wetzlar, Germany) four days after injection.

## 3. Results

### 3.1. Identification and Phylogenetic Analysis of Potato LRR-RLKs

In previous studies, 213, 309 and 234 *LRR-RLK* genes had been identified in *Arabidopsis*, rice, and tomato genomes, respectively [3,32]. To identify *LRR-RLK* genes in potato, a BLASTP search was employed using the full-length sequence of the reported *Arabidopsis* members as queries. Further, Pfam and SMART analyses were performed to ensure the presence of both the LRR and RLK domains in each candidate. As a result, a total of 246 *LRR-RLK* genes were identified in the *S. tuberosum* genome. These newly identified members were given names with the prefix ‘St’ indicating *S. tuberosum*, followed by numbers designated based on their positions from top to bottom on the 12 chromosomes. Detailed information of the StLRR-RLK family genes, including the accession numbers and their characteristics is given in Supplementary file S2.

**Figure 1:**
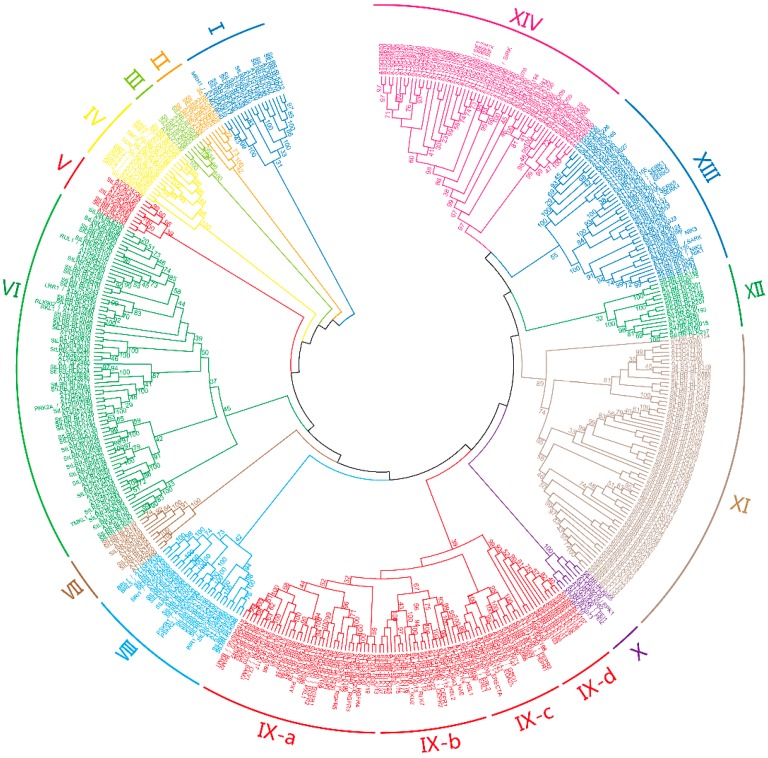
Phylogenetic analysis of LRR-RLK members. The phylogenetic tree was generated from the alignment result of the full-length amino acid sequences by the neighbor-joining (NJ) method. All StLRR-RLK members, together with their *Arabidopsis* homologues, were classified into 14 distinct subfamilies.

To investigate the phylogenetic relationship between the LRR-RLK members from potato and their reported *Arabidopsis* homologues, a neighbor-joining tree was constructed based on the multiple sequence alignment of the full-length sequences. The StLRR-RLKs, together with the members from *Arabidopsis*, were grouped into 14 subfamilies (Figure 1). Each subfamily harbored LRR-RLK members from both potato and *Arabidopsis*. In general, the number of *StLRR-RLK* genes was similar to the number of members from *Arabidopsis* within same subfamily, except for subfamilies XI and XIV. The summarized information regarding each subfamily was listed in Table 1.

The subfamily IX represented the second largest subfamily, and it was further categorized into four subgroups. The well-known LRR-RLK members in *Arabidopsis* including CLV1, BAM1, BAM2, PXY, and RGFRs were clustered within subgroup IX-a. Further, the *Arabidopsis* HAESA, HSL2, and CEPRs fell into subgroup IX-b, while ERECTA, ERLs, and PEPRs were grouped within subgroup IX-c. To gain further insight into the phylogenetic relationships, a number of LRR-RLK members from rice were used to perform a broader analysis. The selected rice members have been previously identified as being homologous to CLV1, BAMs, RGFR, HAESA, and CEPRs [3]. As expected, all of these rice LRR-RLK members fall into subfamily IX, providing further evidence of the phylogenetic relationships between potential peptide-binding receptors (Supplementary file S3). In previous studies, OsCLV1(Osi008429.1), SlCLV1(Solyc04g081590), and PtCLV1(POPTR_0005s26300.1) have been identified as the homologues of AtCLV1 in rice, tomato, and poplar respectively [3,32,33]. In this study, StLRR-RLK117 was grouped together with OsCLV1, SlCLV1, PtCLV1, and AtCLV1, further supporting that StLRR-RLK117 is the homologue of AtCLV1 in potato (Supplementary file S3). Notably, most of the *Arabidopsis* members in subfamily IX were reported to participate in plant growth and developmental processes by interacting with peptide ligands, indicating that their potato homologues may undertake similar functions by perceiving peptide signals.

### 3.2. Conserved and Functional Motifs

Generally, the extracellular LRR domains of LRR-RLK members perceive the ligand and establish the basis of their functions. To unveil the structural diversity and functional characteristics of potato LRR-RLKs, the LRR domains of the identified StLRR-RLK proteins were subjected to motif analysis using the MEME software. In potato, 29 LRR motifs were identified, and their patterns of amino acid sequences were generally found to match with the plant LRR consensus sequence GxIPxxLxxLxxLxxLxLxxNxLx (Supplementary files S4 and S5). The most conserved amino acid residues in potato LRR motifs were Gly at position 1, Pro at position 4, Leu at position 13, 16 and 18, and Asn at position 21. Ile and Leu were at positions 3 and 7 respectively, while Leu and Phe at position 23 were often substituted by each other. In addition, conserved amino acid residues were also found at some other positions of the LRR motifs. These amino acid residues included Pro at position 5 of M14 and M22; Glu at position 6 of M6 and M21; Asn at position 9 of M21, M26 at position 20 of M2. Gly at position 8, Asp at position 17, and Ser at position 19 were also found on a number of LRR motifs (Supplementary file S4).

Generally, the extracellular LRR domains of LRR-RLK members perceive the ligand and establish the basis of their functions. To unveil the structural diversity and functional characteristics of potato LRR-RLKs, the LRR domains of the identified StLRR-RLK proteins were subjected to motif analysis using the MEME software. In potato, 29 LRR motifs were identified, and their patterns of amino acid sequences were generally found to match with the plant LRR consensus sequence GxIPxxLxxLxxLxxLxLxxNxLx (Supplementary files S4 and S5). The most conserved amino acid residues in potato LRR motifs were Gly at position 1, Pro at position 4, Leu at position 13, 16 and 18, and Asn at position 21. Ile and Leu were at positions 3 and 7 respectively, while Leu and Phe at position 23 were often substituted by each other. In addition, conserved amino acid residues were also found at some other positions of the LRR motifs. These amino acid residues included Pro at position 5 of M14 and M22; Glu at position 6 of M6 and M21; Asn at position 9 of M21, M26 at position 20 of M2. Gly at position 8, Asp at position 17, and Ser at position 19 were also found on a number of LRR motifs (Supplementary files S4 and S5).

**Figure 2:**
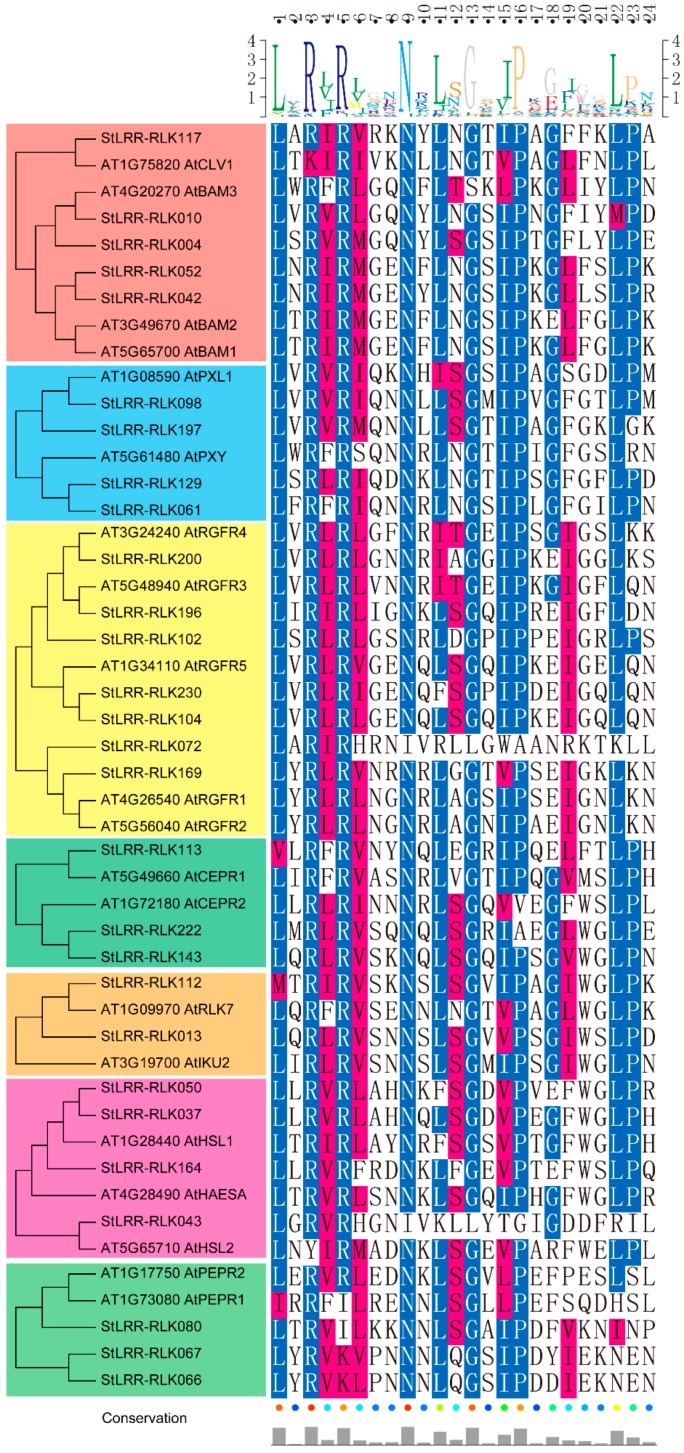
Functional motif analysis of leucine-rich repeat receptor-like kinase (LRR-RLK) members. Sequence alignment of potato and *Arabidopsis* LRR-RLK members harboring the RxR motif among subfamily IX. The highly conserved residues are blue- or red-colored, and are visualized by Texshade (https://ctan.org/pkg/texshade).

Intriguingly, many plant peptides possess histidine or asparagine as their last residue, and the last residue forms salt bridges with the RxR motif (the x stands for any amino acid) in the LRR domain [17]. Including CLV1, BAM1, BAM2, PXY, and RGFRs, the *Arabidopsis* members which were grouped within the subfamily IX all contained such a conserved RxR motif, and all of them have been shown to act as receptors of small peptides. Notably, this RxR motif was found to be largely conserved in the potato members of subfamily IX, suggesting that these potato LRR-RLKs may recognize specific peptide ligands with their RxR motifs (Figure 2).

### 3.3. Exon–Intron Organization

**Figure 3:**
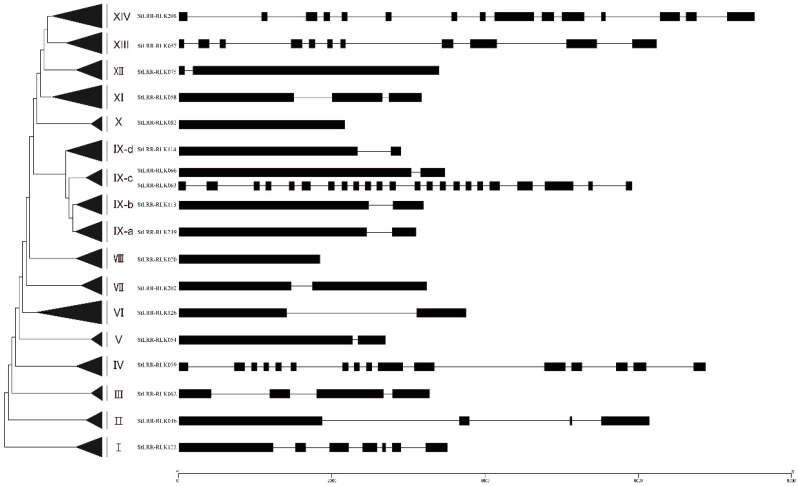
Representative *LRR-RLK* gene structures from each subfamily in potato. The exon–intron organizations of representative *LRR-RLK* genes were visualized by the Gene Structure Display Server (GSDS: http://gsds.cbi.pku.edu.cn/). In each subfamily, the selected gene shares the same structure with the majority of the members. Exons and introns were plotted by boxes and lines, respectively.

The gene structure could provide the evolutional evidence of a gene family. The detailed exon–intron organization for the *StLRR-RLK* genes and their *Arabidopsis* homologues were plotted and listed in Supplementary file S6, and the representative genes from each subfamily were selected and illustrated in Figure 3. Among the 246 *StLRR-RLK* genes, 23 genes had no intron while one, two, three, four, and five introns were found in 118, 29, 14, six, and three genes respectively. In addition, 23 *StLRR-RLK* genes consisted 6~10 introns, 16 *StLRR-RLKs* contained 11~15 introns, while the others possessed more than 15 introns. *StLRR-RLK* genes within the same subgroup were found to share similar exon/intron structures, supporting the results of our phylogenetic analysis. For example, all the members in subfamily XII had one intron, whereas most members in subfamily IV had 15 introns (Supplementary file S6). Interestingly, the intron number of potato *LRR-RLKs* in subfamily IX ranged mostly between 0–2, except for two members, *StLRR-RLK173* and *StLRR-RLK063*, which harbored the maximum number of introns, 21 and 26 respectively.

### 3.4. Chromosome Localization and Duplication Analysis

**Figure 4:**
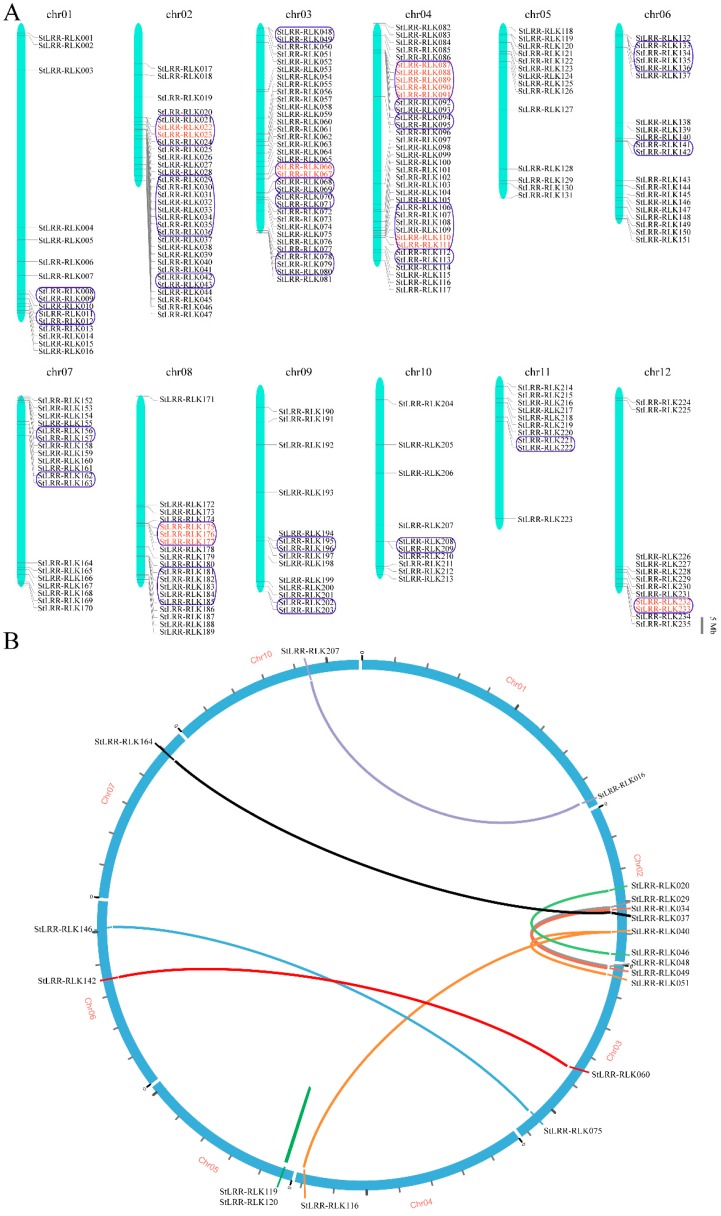
Chromosome distribution and duplication analysis of the potato *LRR-RLK* genes. (**A**) 235 *StLRR-RLK* genes were mapped to 12 potato chromosomes. The blue box indicated a gene cluster and the tandem duplication is indicated by red colored names; (**B**) The 10 putative segmental duplication pairs are linked by the colored lines respectively.

Gene family expansion is an important strategy for the evolutionary fitness of plants. To identify gene clusters and to investigate such events, first we mapped the physical distributions of the *StLRR-RLK* genes, and then these genes were grouped into a cluster if they were arranged in a region within 200 kb (Figure 4A). 235 of the 246 *StLRR-RLK* genes were distributed across 12 potato chromosomes, and a total of 74 *StLRR-RLK* genes were grouped into 26 clusters over 11 chromosomes, with no cluster on chromosome 5, and the maximum of five clusters each on chromosome 3 and 4. The smallest gene cluster comprised only two genes, while the largest cluster was found to have eight tightly grouped genes on chromosome 2. With 70% sequence similarities over the full-length amino acid sequence as the threshold, tandem duplicated genes were identified in several clusters. Among the 26 clusters, 16 genes from six clusters were presumed to be genes derived from tandem duplication. Furthermore, segmental duplication or whole genome duplication of the *StLRR-RLK* genes was analyzed. Among these potato *LRR-RLK* genes, 10 pairs of genes were predicted to be the results of segmental duplication events (Figure 4B). All the tandem and segmental duplication genes were listed in Supplementary file S7. About 10.5% of these genes may have been involved in tandem or segmental duplication, indicating that gene duplication events may play an important role in the expansion of this family.

### 3.5. The Expression Pattern in Tested Tissues

**Figure 5:**
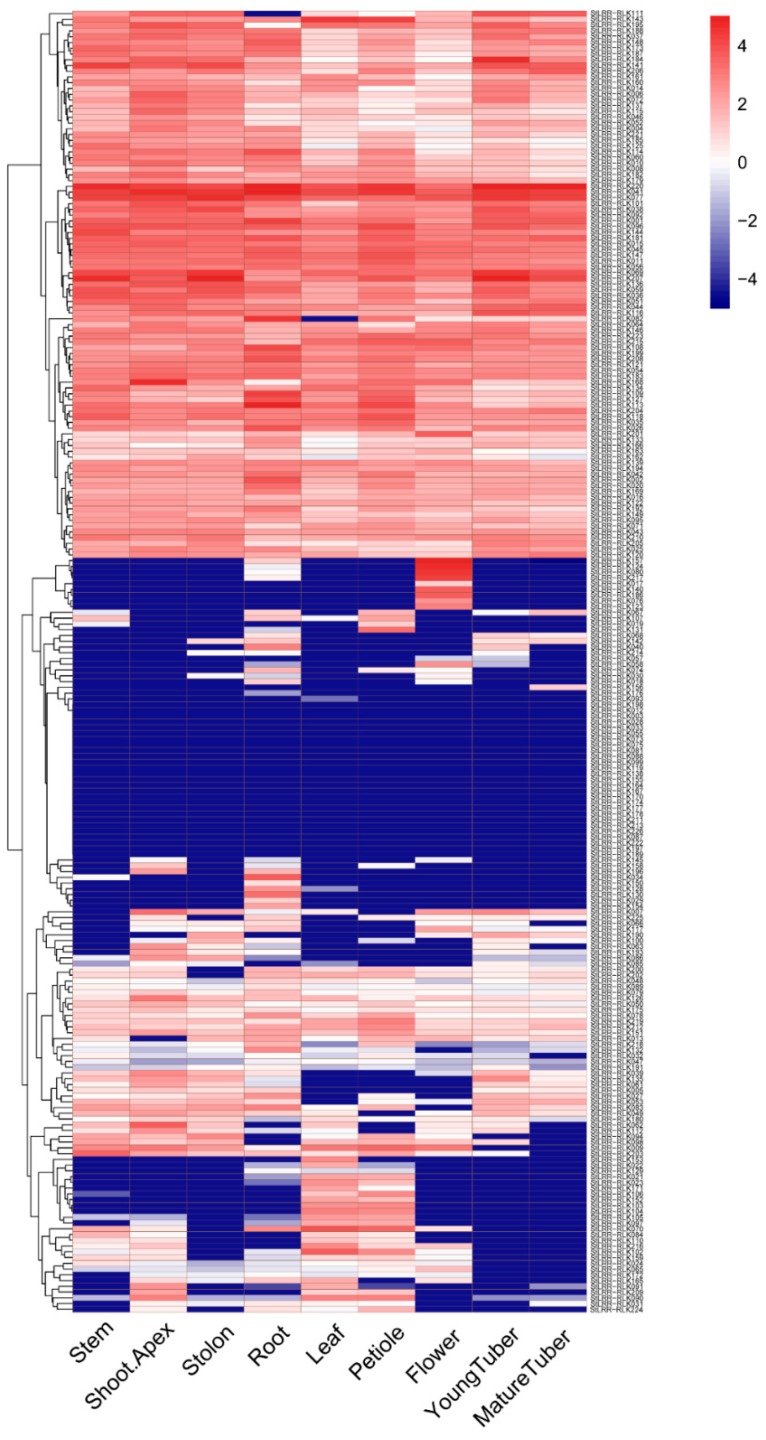
Expression patterns of the potato *LRR-RLK* family genes. The expression pattern data were normalized and visualized by R.

To understand the potential functions of the identified *StLRR-RLK* genes, expression patterns of these genes were analyzed using the RNA-seq data retrieved from PGSC [40]. Transcription levels of the 246 *StLRR-RLK* genes in eight tested tissues including stem, shoot apex, stolon, root, leaf, petiole, flower, and young and mature tuber, were analyzed and visualized. Generally, these genes were found to be expressed broadly in different tissues with considerable variations between individual genes. Some genes exhibited tissue specific expression patterns. For example, *StLRR-RLK017*, *StLRR-RLK 076*, and *StLRR-RLK086* were found to be only expressed in the flower (Figure 5).

The gene expression patterns provided preliminary clues for their functions. For example, *StLRR-RLK117* showed abundant expression in shoot apex, stolon, and flower, implying that *StLRR-RLK117* might be important for the shoot apical meristem, similar to *AtCLV1*. Similar to the expression pattern of *AtBAMs*, potato BAM homologues *StLRR-RLK042*, *StLRR-RLK052*, *StLRR-RLK004* and *StLRR-RLK010* were found to be highly expressed in all tested tissues. Interestingly, *PXY* has been reported to be highly expressed in procambial cells of related organs, including the root and stem in *Arabidopsis*. Further, *StLRR-RLK061* and *StLRR-RLK129* were found to be the homologues of *Arabidopsis PXY*, and they exhibited different expression patterns. *StLRR-RLK061* was found to express in the shoot, stolon, and tuber, whereas *StLRR-RLK129* was expressed in the root and leaf, indicating that functional divergence may have occurred in these two homologues.

### 3.6. Expression Profiling of StLRR-RLK Genes during Abiotic and Biotic Stresses

LRR-RLKs also act as surface receptors, playing vital roles in perceiving and transducing stress-related signals. To analyze the stress responsiveness of the *StLRR-RLK* genes, we compared the transcription levels of *StLRR-RLK* genes under different stresses. Abiotic stress treatments included salt, drought, and heat. Biotic stress treatments included *P. infestans* inoculation and treatments of two chemical elicitors. The relative expression changes of various *StLRR-RLKs* in response to each treatment were calculated in comparison with the respective controls.

**Figure 6:**
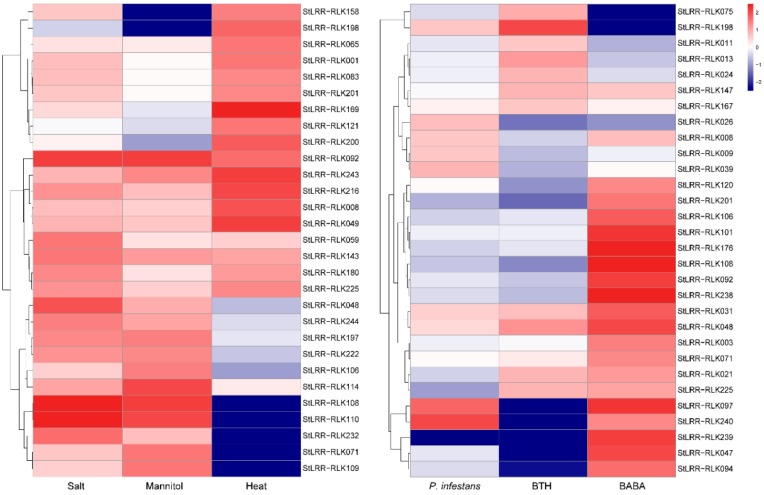
Heat map representation and hierarchical clustering of selected *StLRR-RLK* genes under abiotic and biotic stresses. The relative expression ratios of biotic and abiotic stress treatments were calculated relative to their controls respectively, and then up-regulated genes were defined to have a log_2_ relative expression ratio ≥1 under certain stress treatments. The bars of heat map represent the log_2_ relative expression ratio. The red color, white color, and blue color represent the up-regulated, unaltered, and down-regulated expression respectively.

29 *StLRR-RLK* genes were induced under at least one of the abiotic stress treatments (Figure 6). Out of these 29 genes, *StLRR-RLK008*, *StLRR-RLK049*, *StLRR-RLK092*, *StLRR-RLK143*, *StLRR-RLK216*, *StLRR-RLK225*, and *StLRR-RLK243* displayed multiple stress responses. Several *StLRR-RLKs* exhibited differential expression under various stress treatments. For example, *StLRR-RLK048*, *StLRR-RLK071*, *StLRR-RLK108*, *StLRR-RLK110*, *StLRR-RLK114*, *StLRR-RLK197*, *StLRR-RLK222*, *StLRR-RLK 232*, and *StLRR-RLK244* were induced by both salt stress and drought treatments, while *StLRR-RLK001*, *StLRR-RLK083*, *StLRR-RLK158*, *StLRR-RLK180*, and *StLRR-RLK201* were induced under salt and heat stress conditions. Some of the *StLRR-RLKs* also exhibited enhanced expression under specific stress treatments. For example, *StLRR-RLK059* was up-regulated only under salt stress, while *StLRR-RLK106* and *StLRR-RLK109* were induced under drought treatment only.

To investigate the expression changes of *StLRR-RLKs* under biotic stress treatments, detached leaves of potato plants were treated with *P. infestans* inoculum (Pi) and two chemical elicitors, DL-β-amino-n-butyric acid (BABA, 2 mg/mL) and acibenzolar-s-methyl (BTH, 100 μg/mL). Samples were collected at 24 h, 48 h and 72 h after treatments. A total of 30 *StLRR-RLKs* were detected to be up-regulated under at least one biotic stress treatment (Figure 6). Interestingly, *StLRR-RLK031* was induced under all biotic stress conditions, while the expression of *StLRR-RLK008*, *StLRR-RLK097*, and *StLRR-RLK240* were induced under both *P. infestans* infection and BTH treatment but down-regulated under BABA treatment. The expression of *StLRR-RLK021*, *StLRR-RLK048*, *StLRR-RLK147*, and *StLRR-RLK225* were induced under both BABA and BTH treatments while *StLRR-RLK198* was induced only under *P. infestans* infection and the BABA treatment. The expression of *StLRR-RLK009*, *StLRR-RLK026*, and *StLRR-RLK039* were induced only under *P. infestans* infection, *StLRR-RLK011*, *StLRR-RLK013*, *StLRR-RLK024*, and *StLRR-RLK075* were specifically induced by BABA treatment and *StLRR-RLK003*, *StLRR-RLK047*, *StLRR-RLK071*, *StLRR-RLK092*, *StLRR-RLK094*, *StLRR-RLK101*, *StLRR-RLK106*, *StLRR-RLK108*, *StLRR-RLK120*, and *StLRR-RLK176* were induced under BTH treatment only.

### 3.7. Validation of Expression Patterns by qRT-PCR

**Figure 7:**
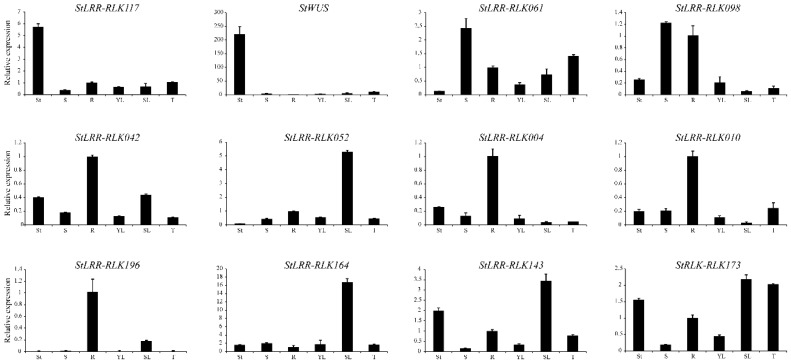
Transcriptional level of selected *StLRR-RLK* genes in various tissues by qRT-PCR. To reveal the tissue specificity, the expression level of the genes was represented as folds relative to the expression level of root. These qRT-PCR data presented here were obtained from three independent biological replicates with three technical repeats. St, shoot tip; S, stem; R, root; YL, young leaf; SL, senescence leaf; T, tuber.

To validate the expression changes of the *StLRR-RLK* genes, a number of representative *StLRR-RLK* genes were selected to perform qRT-PCR analysis. As shown in Figure 7, the qRT-PCR results of representative genes were found to be in agreement with the RNA-seq analysis in general. The minor differences could be due to the difference in samples collection at different developmental stages.

Notably, *StLRR-RLK117* was found to be only expressed in shoot tips within the tested tissues, which strongly suggested that this gene may undertake conserved function as *AtCLV1*. Considering that CLV1 could mediate the CLV3 peptide signal to confine *WUS* expression in *Arabidopsis* and *StWUS* had been identified in previous studies [42], the expression pattern of *StWUS* was also analyzed. The results showed that *StLRR-RLK117* shares a similar expression pattern with *StWUS* (Figure 7).

### 3.8. Bimolecular Fluorescence Complementation Assays

**Figure 8:**
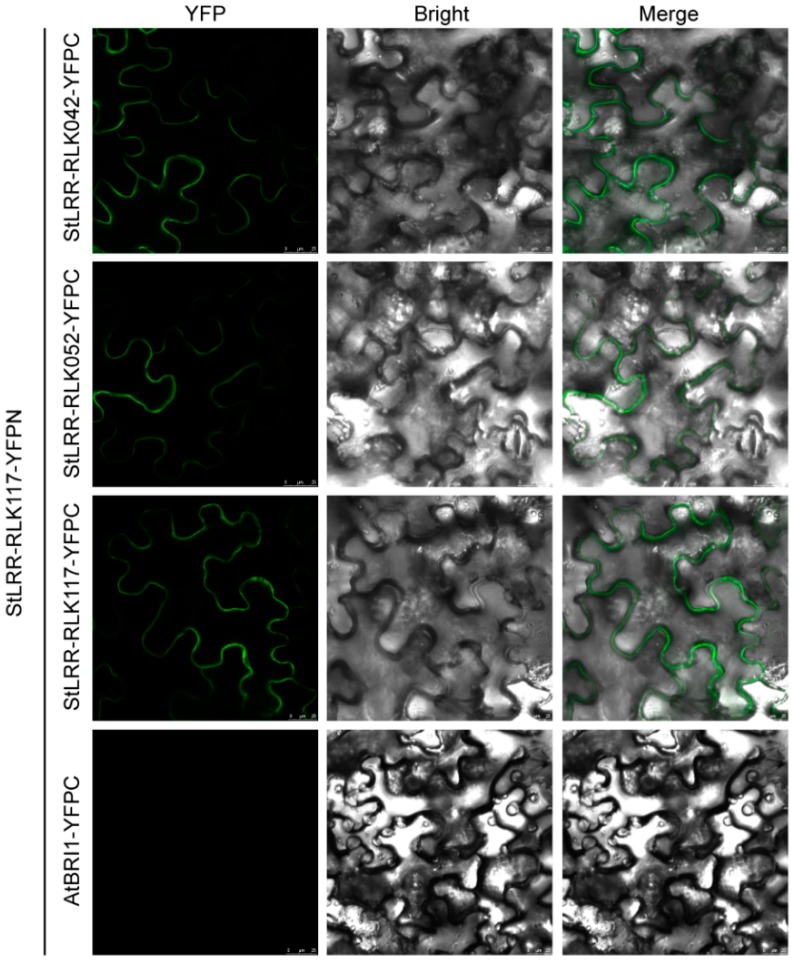
BiFC assay in *N. benthamiana* showing the interactions of StLRR-RLK117 with StLRR-RLK042, StLRR-RLK052, and StLRR-RLK117 respectively. In this study, StLRR-RLK117 was fused to the N-terminal part of YFP while StLRR-RLK042, StLRR-RLK052, StLRR-RLK117, and AtBRI1 were fused to the C-terminal part of YFP respectively. Scale bars, 25 µm.

In previous studies, AtCLV1 was observed to interact with AtBAM1, AtBAM2, and AtCLV1 itself, and to transduce the CLV3 peptide signal by forming homodimers and heterodimers in regulating stem cell homeostasis [9]. To explore such molecular mechanisms in potato, bimolecular fluorescence complementation (BiFC) was used to analyze the interactions between the selected potato LRR-RLK receptors. In this study, StLRR-RLK117, which is the homologue of AtCLV1, was fused to the N-terminal fragment of YFP, while StLRR-RLK042, StLRR-RLK052, and StLRR-RLK117 itself were fused to the C-terminal fragment of YFP. In the case that the two tested proteins associated with each other, a full complex of fluorescent YFP would form and be detected. Notably, YFP fluorescence was observed on the membrane when injecting StLRR-RLK117-YFPN together with StLRR-RLK042-YFPC, StLRR-RLK052-YFPC, or StLRR-RLK117-YFPC, but not with AtBRI1-YFPC (Figure 8). Considering that StLRR-RLK042 and StLRR-RLK052 were grouped with AtBAM1 and AtBAM2, these results indicated that potato CLV1 and BAMs may also interact with each other, and StLRR-RLK117 could also form homodimers in regulating stem cell homeostasis in the SAM.

## 4. Discussion

In this study, a comprehensive analysis of the LRR-RLK family in potato was carried out, and 246 *StLRR-RLK* genes were identified and analyzed. Based on the phylogenetic analysis, StLRR-RLK proteins together with reported *Arabidopsis* members fell into 14 subfamilies, and each subfamily contained members from both potato and *Arabidopsis*, implying that the divergence of the *LRR-RLK* gene family may appear before the divergence of these two species. The classifications predicted by phylogenetic analysis were further supported by the similar exon–intron organization and motif arrangement within the same subfamily. 235 *StLRR-RLK* genes were anchored on 12 potato chromosomes, and a total of 16 genes from six clusters were inferred to be tandem duplicated genes. The expression profiling retrieved from RNA-seq and qRT-PCR data in various tissues revealed that *StLRR-RLK* genes showed similar or distinct expression patterns compared with their *Arabidopsis* homologues, implying either functional conservation or divergence. The expression pattern under abiotic/biotic treatments indicated that some *StLRR-RLK* genes also were up-regulated under specific stress treatments, and they may be involved in stress responses.

The subfamily XI represented the largest subfamily, and a significant expansion event of potato LRR-RLK members in this subfamily was observed. 59 LRR-RLK members from potato were found to form a separate branch on the phylogenetic tree (Figure 2). Thirty-four of the 59 members were grouped in clusters on chromosomes, and seven genes were found to come from tandem duplication, while four genes came from segmental duplication. These results suggested that most of the 34 subfamily XI *LRR-RLK* genes might be originated from gene duplication events after the separation of potato and *Arabidopsis*. Similar exon–intron organization and motif arrangement of these members further supported this hypothesis. Furthermore, among the 29 *StLRR-RLK* genes that were significantly up-regulated under at least one of the abiotic stress treatments, eight of them were members of subfamily XI. Similarly, among the 30 *StLRR-RLK* genes that were induced under tested biotic stress treatments, 10 members were from this subfamily. These results indicated that the gene expansion events within subfamily XI during evolution may help in sensing diverse stress signals in potato.

For subfamily IX, some well-studied LRR-RLK members in *Arabidopsis*, including CLV1, BAM1, BAM2, PXY, HAESA, HSL2, ERECTA, PEPRs, CEPRs, and RGFRs were clustered within this subfamily. Interestingly, all of these LRR-RLK receptors were reported to play important roles in development, plant growth, and stress responses, by interacting with peptide ligands, implying that the potato members in this subfamily may also bind peptide ligands. Furthermore, the RxR motif is conserved in all potato members of subfamily IX, enabling these members to interact with peptide ligands. In addition, exon–intron organization analysis revealed that most of the members in subfamily IX possessed one intron, whereas ERECTA, ERL1, and ERL2, together with their close potato homologue StLRR-RLK173 and StLRR-RLK063, contained over 20 introns, supporting the results of the phylogenetic analysis. Interestingly, the intron-less *ERECTA* gene could not rescue the phenotype of the *erecta* mutants, indicating that the function of *ERECTA* depends on the presence of introns [43]. Considering the conservation of gene structures, the introns of *StLRR-RLK173* and *StLRR-RLK063* may also be essential to their functions. Notably, In *Arabidopsis*, *PXY* was found to be highly expressed in procambial cells of related organs, including the root and stem. Moreover, *TDIF-PXY-WOX4* signaling was reported to play an important role in cell fate decisions of the vascular meristem [11,14]. Consistently, *StLRR-RLK061* proved to serve as the homologue of *PXY* and exhibited high expression in the shoot, shoot apex, stolon, young tuber, and mature tuber, suggesting that this gene might be critical in the regulation of vascular organization in the shoot. Interestingly, tubers are swollen stem structures that are used as storage organs. Considering its higher expression in tuber tissue, *StLRR-RLK061* may participate in the formation of tubers, and may affect the yield of potato. Further, BiFC assays revealed that StLRR-RLK117 may interact with StLRR-RLK042 and StLRR-RLK052, while StLRR-RLK117 could also form homodimers. The preferred expression of *StLRR-RLK117*, which is the homologue of *AtCLV1*, in the shoot apex, implied that this gene may also be involved in the development of the SAM. The *CLV3-CLV1/BAM-WUS* feedback loop was reported to be important in shoot apical stem-cell maintenance activity, and *StWUS* had already been identified in a previous study [42]. The overlap expression pattern of *StWUS* and *StLRR-RLK117* suggested that this mechanism could be conserved in potato. It was supposed that *StWUS* may be suppressed by StLRR-RLK117, StLRR-RLK042, and StLRR-RLK052 after perceiving certain peptide signals.

## 5. Conclusions

In this study, a systematic study was carried out to identify and characterize the LRR-RLK family genes in potato. The potato LRR-RLK family genes were studied through phylogeny, motif, gene structure, chromosomal distribution, and expression profiling analysis, which provides insight into the evolutionary conservation of this gene family. Notably, the positive result of the BiFC assay and the overlapping expression region between *StLRR-RLK117* and *StWUS* indicated that the *CLV3-CLV1/BAM-WUS* feedback loop may be conserved in maintaining stem cell homeostasis within the potato shoot apical meristem.

## Figures and Tables

**Table 1:** Subfamily designation and sequence characteristics of the identified *StLRR-RLK* genes.

Subfamily	Gene Num.	PI	MW (kDa)	Amino Acid Length	SP (%)	*Arabidopsis* Best Hits
I	9	5.48–9.18	59.6–87.0	533–785	77.8	*MRH1*
II	4	6.14–8.87	61.6–105.4	561–959	100	
III	3	5.88–8.12	74.0–75.4	678–686	100	
IV	5	5.62–6.26	75.0–80.8	692–738	80	*SRF1-8*, *SCM*
V	4	6.26–9.35	76.4–105.3	682–946	100	
VI	41	5.69–9.30	63.9–122.0	565–1127	75.6	*RUL1*, *LRR1*, *RLK902*, *RKL1*, *PRK2A*, *TMKL1*
VII	3	5.36–6.44	95.8–107.4	882–984	33.3	
VIII	15	5.2–8.9	41.3–137.8	369–1272	66.7	*BRL1*, *BRL3*, *BRI1*, *PSRK1*, *PSKR2*, *BIR1*
IX-a	20	5.14–8.21	99.6–133.3	919–1230	80	*CLV1*, *BAM1-3*, *PXY*, *PXL1*, *RGFR1-5*
IX-b	16	5.02–8.99	60.6–114.6	542–1032	87.5	*HAE*, *HSL1-2*, *CEPR1-2*, *RLK7*
IX-c	8	5.12–9.37	70.2–138.1	625–1255	100	*PEPR1-2*, *GSO1*, *ERECTA*, *ERL1-2*
IX-d	10	5.18–9.03	88.7–141.0	804–1283	70	
X	3	5.43–6.87	67.0–121.7	601–1126	66.7	*FEI1*, *FEI2*
XI	60	5.11–8.83	33.8–222.9	303–2007	41.7	
XII	10	5.38–6.64	10.4–121.8	921–1106	90	
XIII	16	5.43–8.4	57.6–113.3	518–1027	62.5	*SERK1-5*, *SARK*, *NIK1-3*
XIV	10	5.76–8.75	57.4–106.0	518–964	70	*SIF1-4*, *SIRK*

Note: PI, isoelectric point; MW, molecular weight; SP, signal peptide.

## Supplementary Materials

The following are available online at http://www.mdpi.com/2073-4409/7/9/120/s1, Supplementary file S1: The qRT-PCR primers used in this study, Supplementary file S2: The information of identified potato LRR-RLK family members, Supplementary file S3: Phylogenetic analysis of LRR-RLK members in potato, rice, and *Arabidopsis*, Supplementary file S4: LRR motif analysis in the LRR domain of identified potato LRR-RLK members, Supplementary file S5: The conserved LRR motifs of StLRR-RLK members as identified by MEME, Supplementary file S6: The exon/intron structures of *StLRR-RLK* genes, Supplementary file S7: The identified gene cluster and duplication analysis of *StLRR-RLK* genes.

## References

[1] Gish L.A., Clark S.E. (2011). The RLK/Pelle family of kinases. Plant J..

[2] Shiu S.H., Bleecker A.B. (2001). Receptor-like kinases from *Arabidopsis* form a monophyletic gene family related to animal receptor kinases. Proc. Natl. Acad. Sci. USA.

[3] Shiu S.H., Karlowski W.M., Pan R., Tzeng Y.H., Mayer K.F., Li W.H. (2004). Comparative analysis of the receptor-like kinase family in *Arabidopsis* and rice. Plant Cell.

[4] Clark S.E., Williams R.W., Meyerowitz E.M. (1997). The *CLAVATA1* gene encodes a putative receptor kinase that controls shoot and floral meristem size in *Arabidopsis*. Cell.

[5] Clark S.E., Running M.P., Meyerowitz E.M. (1993). *CLAVATA1*, a regulator of meristem and flower development in *Arabidopsis*. Development.

[6] Schoof H., Lenhard M., Haecker A., Mayer K.F., Jürgens G., Laux T. (2000). The stem cell population of *Arabidopsis* shoot meristems is maintained by a regulatory loop between the *CLAVATA* and *WUSCHEL* genes. Cell.

[7] Clark S.E. (2001). Cell signalling at the shoot meristem. Nat. Rev. Mol. Cell Biol..

[8] DeYoung B.J., Bickle K.L., Schrage K.J., Muskett P., Patel K., Clark S.E. (2006). The CLAVATA1-related BAM1, BAM2 and BAM3 receptor kinase-like proteins are required for meristem function in *Arabidopsis*. Plant J..

[9] Guo Y., Han L., Hymes M., Denver R., Clark S.E. (2010). CLAVATA2 forms a distinct CLE-binding receptor complex regulating *Arabidopsis* stem cell specification. Plant J..

[10] Hu C., Zhu Y., Cui Y., Cheng K., Liang W., Wei Z., Zhu M., Yin H., Zeng L., Xiao Y. (2018). A group of receptor kinases are essential for *CLAVATA* signalling to maintain stem cell homeostasis. Nat. Plants.

[11] Hirakawa Y., Kondo Y., Fukuda H. (2010). TDIF peptide signaling regulates vascular stem cell proliferation via the WOX4 homeobox gene in *Arabidopsis*. Plant Cell.

[12] Zhang H., Lin X., Han Z., Qu L.J., Chai J. (2016). Crystal structure of PXY-TDIF complex reveals a conserved recognition mechanism among CLE peptide-receptor pairs. Cell Res..

[13] Kondo Y., Fukuda H. (2015). The TDIF signaling network. Curr. Opin. Plant Biol..

[14] Fisher K., Turner S. (2007). PXY, a receptor-like kinase essential for maintaining polarity during plant vascular-tissue development. Curr. Biol..

[15] Stenvik G.E., Tandstad N.M., Guo Y., Shi C.-L., Kristiansen W., Holmgren A., Clark S.E., Aalen R.B., Butenko M.A. (2008). The EPIP peptide of INFLORESCENCE DEFICIENT IN ABSCISSION is sufficient to induce abscission in *Arabidopsis* through the receptor-like kinases HAESA and HAESA-LIKE2. Plant Cell.

[16] Tabata R., Sumida K., Yoshii T., Ohyama K., Shinohara H., Matsubayashi Y. (2014). Perception of root-derived peptides by shoot LRR-RKs mediates systemic N-demand signaling. Science.

[17] Song W., Liu L., Wang J., Wu Z., Zhang H., Tang J., Lin G., Wang Y., Wen X., Li W. (2016). Signature motif-guided identification of receptors for peptide hormones essential for root meristem growth. Cell Res..

[18] Matsuzaki Y., Ogawa-Ohnishi M., Mori A., Matsubayashi Y. (2010). Secreted peptide signals required for maintenance of root stem cell niche in *Arabidopsis*. Science.

[19] Lee J.S., Hnilova M., Maes M., Lin Y.C.L., Putarjunan A., Han S.-K., Avila J., Torii K.U. (2015). Competitive binding of antagonistic peptides fine-tunes stomatal patterning. Nature.

[20] He Z., Wang Z.Y., Li J., Zhu Q., Lamb C., Ronald P., Chory J. (2000). Perception of brassinosteroids by the extracellular domain of the receptor kinase BRI1. Science.

[21] Kinoshita T., Cano-Delgado A., Seto H., Hiranuma S., Fujioka S., Yoshida S., Chory J. (2005). Binding of brassinosteroids to the extracellular domain of plant receptor kinase BRI1. Nature.

[22] Huffaker A., Pearce G., Ryan C.A. (2006). An endogenous peptide signal in *Arabidopsis* activates components of the innate immune response. Proc. Natl. Acad. Sci. USA.

[23] Yamaguchi Y., Huffaker A., Bryan A.C., Tax F.E., Ryan C.A. (2010). PEPR2 is a second receptor for the Pep1 and Pep2 peptides and contributes to defense responses in *Arabidopsis*. Plant Cell.

[24] Chinchilla D., Bauer Z., Regenass M., Boller T., Felix G. (2006). The *Arabidopsis* receptor kinase FLS2 binds flg22 and determines the specificity of flagellin perception. Plant Cell.

[25] Sun Y., Li L., Macho A.P., Han Z., Hu Z., Zipfel C., Zhou J.M., Chai J. (2013). Structural basis for flg22-induced activation of the *Arabidopsis* FLS2-BAK1 immune complex. Science.

[26] Yuan N., Yuan S., Li Z., Zhou M., Wu P., Hu Q., Wang L., Mendu V., Luo H. (2018). STRESS INDUCED FACTOR 2, a leucine-rich repeat kinase regulates basal plant pathogen defense. Plant Physiol..

[27] Osakabe Y., Maruyama K., Seki M., Satou M., Shinozaki K., Yamaguchi-Shinozaki K. (2005). Leucine-rich repeat receptor-like kinase1 is a key membrane-bound regulator of abscisic acid early signaling in *Arabidopsis*. Plant Cell.

[28] Osakabe Y., Mizuno S., Tanaka H., Maruyama K., Osakabe K., Todaka D., Fujita Y., Kobayashi M., Shinozaki K., Yamaguchi-Shinozaki K. (2010). Overproduction of the membrane-bound receptor-like protein kinase1, RPK1, enhances abiotic stress tolerance in *Arabidopsis*. J. Biol. Chem..

[29] Lee I.C., Hong S.W., Whang S.S., Lim P.O., Nam H.G., Koo J.C. (2011). Age-dependent action of an ABA-inducible receptor kinase, RPK1, as a positive regulator of senescence in *Arabidopsis* leaves. Plant Cell Physiol..

[30] Law Y.S., Gudimella R., Song B.K., Ratnam W., Harikrishna J.A. (2012). Molecular characterization and comparative sequence analysis of defense-related gene, *oryza rufipogon* receptor-like protein kinase 1. Int. J. Mol. Sci..

[31] Sakamoto T., Deguchi M., Brustolini O.J., Santos A.A., Silva F.F., Fontes E.P. (2012). The tomato RLK superfamily: Phylogeny and functional predictions about the role of the LRRII-RLK subfamily in antiviral defense. BMC Plant Biol..

[32] Wei Z., Wang J., Yang S., Song Y. (2015). Identification and expression analysis of the *LRR-RLK* gene family in tomato (*Solanum lycopersicum*) Heinz 1706. Genome.

[33] Zan Y., Ji Y., Zhang Y., Yang S., Song Y., Wang J. (2013). Genome-wide identification, characterization and expression analysis of populus *leucine-rich repeat receptor-like protein kinase* genes. BMC Genom..

[34] Magalhães D.M., Scholte L.L., Silva N.V., Oliveira G.C., Zipfel C., Takita M.A., De Souza A.A. (2016). LRR-RLK family from two Citrus species: Genome-wide identification and evolutionary aspects. BMC Genom..

[35] Liu S., Zhang C., Chao N., Lu J., Zhang Y. (2018). Cloning, Characterization, and Functional Investigation of *VaHAESA* from *Vitis amurensis* Inoculated with *Plasmopara viticola*. Int. J. Mol. Sci..

[36] Sun J., Li L., Wang P., Zhang S., Wu J. (2017). Genome-wide characterization, evolution, and expression analysis of the *leucine-rich repeat receptor-like protein kinase* (*LRR-RLK*) gene family in Rosaceae genomes. BMC Genom..

[37] Wang Y., Tang H., DeBarry J.D., Tan X., Li J., Wang X., Lee T.H., Jin H., Marler B., Guo H. (2012). MCScanX: A toolkit for detection and evolutionary analysis of gene synteny and collinearity. Nucleic Acids Res..

[38] Cao Y., Meng D., Chen Y., Abdullah M., Jin Q., Lin Y., Cai Y. (2018). Comparative and Expression Analysis of Ubiquitin Conjugating Domain-Containing Genes in Two *Pyrus* Species. Cells.

[39] Krzywinski M.I., Schein J.E., Birol I., Connors J., Gascoyne R., Horsman D., Jones S.J., Marra M.A. (2009). Circos: An information aesthetic for comparative genomics. Genome Res..

[40] Consortium P.G.S. (2011). Genome sequence and analysis of the tuber crop potato. Nature.

[41] Nicot N., Hausman J.F., Hoffmann L., Evers D. (2005). Housekeeping gene selection for real-time RT-PCR normalization in potato during biotic and abiotic stress. J. Exp. Bot..

[42] Li X., Hamyat M., Liu C., Salman A., Gao X., Guo C., Wang Y., Guo Y. (2018). Identification and Characterization of the WOX Family Genes in Five *Solanaceae* Species Reveal Their Conserved Roles in Peptide Signaling. Genes.

[43] Karve R., Liu W., Willet S.G., Torii K.U., Shpak E.D. (2011). The presence of multiple introns is essential for *ERECTA* expression in *Arabidopsis*. RNA.

