# Supplementary material for: Identification and Characterization of LRR-RLK Family Genes in Potato Reveal Their Involvement in Peptide Signaling of Cell Fate Decisions and Biotic/Abiotic Stress Responses
Source: Cells. 2018 Aug 27;7(9):120. doi: 10.3390/cells7090120 (PMC6162732; doi:10.3390/cells7090120)
Supplement: Supplementary file 1 [file cells-07-00120-s001.zip › Sup File 2.pdf]

Names	Gene ID	pI	Mw (kDa)	ORF (bp)	Protein Length (aa)	Group	Siginal Peptide
StLRR-RLK001	PGSC0003DMT400055038	5.76	10444.29	2868	955	Ⅻ	√
StLRR-RLK002	PGSC0003DMT400042178	6.27	112158.66	3042	1013	Ⅷ	√
StLRR-RLK003	PGSC0003DMT400002647	7.75	132546.48	3603	1200	Ⅺ	√
StLRR-RLK004	PGSC0003DMT400016060	7.22	105911.52	2883	960	Ⅸ	√
StLRR-RLK005	PGSC0003DMT400046249	8.93	70435.07	1914	637	Ⅰ	√
StLRR-RLK006	PGSC0003DMT400066895	8.12	75443.54	2061	686	Ⅲ	√
StLRR-RLK007	PGSC0003DMT400070722	8.34	85809.46	2337	778	Ⅰ	√
StLRR-RLK008	PGSC0003DMT400047024	6.31	93350.49	2547	848	ⅩⅣ	√
StLRR-RLK009	PGSC0003DMT400047023	5.9	104893.65	2844	947	ⅩⅣ	√
StLRR-RLK010	PGSC0003DMT400064038	6.17	109937.31	3000	999	Ⅸ	√
StLRR-RLK011	PGSC0003DMT400032797	5.43	57565.74	1557	518	ⅩⅢ	
StLRR-RLK012	PGSC0003DMT400032784	6.59	72266.42	1965	654	Ⅵ	√
StLRR-RLK013	PGSC0003DMT400013077	5.46	108078	2946	981	Ⅸ	√
StLRR-RLK014	PGSC0003DMT400066225	5.7	84579.17	2316	771	Ⅵ	√
StLRR-RLK015	PGSC0003DMT400066706	5.7	101772.18	2829	942	Ⅻ	√
StLRR-RLK016	PGSC0003DMT400001921	8.42	95873.66	2646	881	Ⅱ	√
StLRR-RLK017	PGSC0003DMT400008229	5.48	59599.29	1602	533	Ⅰ	
StLRR-RLK018	PGSC0003DMT400046038	5.98	120786.63	3288	1095	Ⅺ	
StLRR-RLK019	PGSC0003DMT400026965	5.54	78155.21	2118	705	Ⅺ	
StLRR-RLK020	PGSC0003DMT400017876	8.9	67805.39	1839	612	Ⅷ	√
StLRR-RLK021	PGSC0003DMT400026802	7.44	135400	3696	1231	Ⅺ	
StLRR-RLK022	PGSC0003DMT400026813	5.85	110318.24	2997	998	Ⅺ	
StLRR-RLK023	PGSC0003DMT400026824	6.12	119033.39	3249	1082	Ⅺ	√
StLRR-RLK024	PGSC0003DMT400026739	8.02	50686.58	1365	454	Ⅺ	
StLRR-RLK025	PGSC0003DMT400054594	5.78	112926.85	3138	1045	Ⅵ	
StLRR-RLK027	PGSC0003DMT400073238	6.22	86049.88	2319	772	ⅩⅢ	
StLRR-RLK028	PGSC0003DMT400073093	7.56	69487.82	1878	625	ⅩⅢ	√
StLRR-RLK029	PGSC0003DMT400073277	5.78	128315.38	3474	1157	Ⅺ	
StLRR-RLK030	PGSC0003DMT400073281	5.8	127905.39	3483	1160	Ⅺ	√
StLRR-RLK031	PGSC0003DMT400073105	6.37	56222.1	1506	501	Ⅺ	√
StLRR-RLK032	PGSC0003DMT400073110	6.41	124554.78	3390	1129	Ⅺ	
StLRR-RLK033	PGSC0003DMT400073117	6.43	59997.2	1629	542	Ⅺ	
StLRR-RLK034	PGSC0003DMT400073121	5.8	50702.53	1362	453	Ⅺ	
StLRR-RLK035	PGSC0003DMT400073122	6.59	125873.07	3432	1143	Ⅺ	√
StLRR-RLK036	PGSC0003DMT400073289	9.35	76357.88	2049	682	Ⅴ	√
StLRR-RLK037	PGSC0003DMT400081228	5.02	109729.76	3003	1000	Ⅸ	√
StLRR-RLK038	PGSC0003DMT400057768	6.15	80821.61	2217	738	Ⅳ	√
StLRR-RLK039	PGSC0003DMT400043075	6.26	78531.17	2175	724	Ⅳ	
StLRR-RLK040	PGSC0003DMT400009133	5.84	122036.33	3384	1127	Ⅵ	√
StLRR-RLK041	PGSC0003DMT400078790	8.22	82417.14	2190	729	ⅩⅣ	√
StLRR-RLK042	PGSC0003DMT400078706	6	111377.62	3054	1017	Ⅸ	√

StLRR-RLK043	PGSC0003DMT400078707	6.31	109316.02	2970	989	IX	√
StLRR-RLK044	PGSC0003DMT400064235	6.4	83530.7	2307	768	VI	√
StLRR-RLK045	PGSC0003DMT400026062	8.2	73829.36	1995	664	XIII	√
StLRR-RLK046	PGSC0003DMT400003278	7.08	66231.82	1806	601	VIII	√
StLRR-RLK047	PGSC0003DMT400052237	6.75	84708.29	2304	767	I	√
StLRR-RLK048	PGSC0003DMT400068787	6.4	132446.59	3615	1204	XI	√
StLRR-RLK049	PGSC0003DMT400068782	6.23	94319.89	2532	843	XI	
StLRR-RLK050	PGSC0003DMT400033367	5.28	109127.12	2988	995	IX	√
StLRR-RLK051	PGSC0003DMT400058132	5.69	122013.01	3369	1122	VI	√
StLRR-RLK052	PGSC0003DMT400043020	6.52	112033.93	3069	1022	IX	√
StLRR-RLK053	PGSC0003DMT400033662	7.62	68532.55	1884	627	VI	√
StLRR-RLK054	PGSC0003DMT400033657	8.55	96013.96	2625	874	V	√
StLRR-RLK055	PGSC0003DMT400032190	6.59	121647.08	3381	1126	X	
StLRR-RLK057	PGSC0003DMT400056093	6.66	67426.25	1815	604	XIII	√
StLRR-RLK058	PGSC0003DMT400053483	5.95	94332.52	2577	858	XI	
StLRR-RLK059	PGSC0003DMT400031158	6.51	105214.14	2880	959	XI	
StLRR-RLK060	PGSC0003DMT400013468	7.58	69341.08	1908	635	VI	√
StLRR-RLK061	PGSC0003DMT400048524	6.47	111627.3	3048	1015	IX	
StLRR-RLK062	PGSC0003DMT400059219	8.03	74007.77	2037	678	III	√
StLRR-RLK063	PGSC0003DMT400079665	5.64	81860	2247	748	IX	√
StLRR-RLK064	PGSC0003DMT400037057	5.75	137825.63	3819	1272	VIII	√
StLRR-RLK065	PGSC0003DMT400023418	8.3	68070.74	1878	625	VI	√
StLRR-RLK066	PGSC0003DMT400008336	6.41	123402.66	3348	1115	IX	√
StLRR-RLK067	PGSC0003DMT400008335	6.68	125019.36	3384	1127	IX	√
StLRR-RLK068	PGSC0003DMT400039527	6.81	117480.01	3189	1062	XI	
StLRR-RLK069	PGSC0003DMT400039509	5.94	68721.04	1827	608	VI	√
StLRR-RLK070	PGSC0003DMT400039213	8.01	71704.99	1980	659	VI	√
StLRR-RLK071	PGSC0003DMT400039198	9.37	71339.87	1917	638	IX	√
StLRR-RLK072	PGSC0003DMT400046807	5.75	120304.79	3324	1107	IX	√
StLRR-RLK073	PGSC0003DMT400065173	5.43	66998.64	1806	601	X	√
StLRR-RLK074	PGSC0003DMT400062915	8.53	67452.33	1815	604	VI	
StLRR-RLK075	PGSC0003DMT400063400	6.19	119818.03	3282	1093	XII	√
StLRR-RLK076	PGSC0003DMT400006692	5.94	87970.11	2370	789	XIV	√
StLRR-RLK077	PGSC0003DMT400058948	6.85	74635.62	2028	675	VI	
StLRR-RLK078	PGSC0003DMT400023732	5.89	76888.94	2136	711	IV	√
StLRR-RLK079	PGSC0003DMT400023733	5.8	119549.78	3267	1088	VIII	
StLRR-RLK080	PGSC0003DMT400023681	6.29	120962.98	3315	1104	IX	√
StLRR-RLK081	PGSC0003DMT400023776	6.36	79588.67	2178	725	VI	
StLRR-RLK082	PGSC0003DMT400045887	6.87	80038.08	2166	721	X	√
StLRR-RLK083	PGSC0003DMT400007454	6.99	68835.02	1863	620	XIII	√
StLRR-RLK084	PGSC0003DMT400075691	5.79	122615.25	3372	1123	VIII	√
StLRR-RLK086	PGSC0003DMT400041350	6.2	125874.07	3399	1132	XI	√
StLRR-RLK087	PGSC0003DMT400060792	5.57	124680.95	3387	1128	XI	√
StLRR-RLK088	PGSC0003DMT400060788	6.37	124662.1	3381	1126	XI	√

StLRR-RLK089	PGSC0003DMT400060777	6.28	122066.88	3318	1105	XI	√
StLRR-RLK090	PGSC0003DMT400060766	6.51	124847.16	3396	1131	XI	√
StLRR-RLK091	PGSC0003DMT400060641	6.16	125477.1	3387	1128	XI	√
StLRR-RLK092	PGSC0003DMT400060712	5.43	86845.15	2358	785	XI	√
StLRR-RLK095	PGSC0003DMT400060535	5.42	60308.26	1647	548	XI	
StLRR-RLK096	PGSC0003DMT400019924	6.14	67129.62	1824	607	XIII	
StLRR-RLK097	PGSC0003DMT400062730	5.11	55653.48	1506	501	XI	
StLRR-RLK098	PGSC0003DMT400010618	6.17	111533.82	3039	1012	IX	√
StLRR-RLK099	PGSC0003DMT400033720	6.02	114817.4	3177	1058	VI	√
StLRR-RLK100	PGSC0003DMT400050728	6.36	132090.37	3621	1206	VIII	√
StLRR-RLK101	PGSC0003DMT400031288	8.39	111298.72	3030	1009	IX	√
StLRR-RLK102	PGSC0003DMT400063787	5.42	115560.62	3162	1053	IX	
StLRR-RLK103	PGSC0003DMT400016283	9.03	92085.19	2487	828	IX	
StLRR-RLK104	PGSC0003DMT400064535	5.99	118922.8	3267	1088	IX	
StLRR-RLK105	PGSC0003DMT400070819	5.74	70037.63	1905	634	XIII	
StLRR-RLK106	PGSC0003DMT400045658	5.96	114370.12	3114	1037	IX	√
StLRR-RLK107	PGSC0003DMT400045667	8.46	88754.17	2415	804	IX	
StLRR-RLK108	PGSC0003DMT400071897	6.98	105507.67	2886	961	IX	√
StLRR-RLK109	PGSC0003DMT400071885	5.43	141000.29	3852	1283	IX	
StLRR-RLK110	PGSC0003DMT400071877	7.6	107738.12	2922	973	IX	√
StLRR-RLK111	PGSC0003DMT400016372	7.37	110998.74	3018	1005	IX	√
StLRR-RLK112	PGSC0003DMT400012744	5.83	108682.13	2955	984	IX	√
StLRR-RLK113	PGSC0003DMT400012733	7.77	106505.14	2868	955	IX	√
StLRR-RLK114	PGSC0003DMT400055412	8.77	90254.9	2457	818	IX	√
StLRR-RLK115	PGSC0003DMT400020520	8.73	74535.74	2016	671	VI	√
StLRR-RLK116	PGSC0003DMT400009520	6	118787.87	3306	1101	VI	
StLRR-RLK117	PGSC0003DMT400025747	6.27	108283.87	2949	982	IX	√
StLRR-RLK118	PGSC0003DMT400019446	7.89	68900.26	1866	621	XIII	√
StLRR-RLK119	PGSC0003DMT400037779	8.42	73603.31	2022	673	VI	√
StLRR-RLK120	PGSC0003DMT400078542	6.18	77668.91	2145	714	VI	√
StLRR-RLK121	PGSC0003DMT400059346	5.12	138082.62	3768	1255	IX	√
StLRR-RLK122	PGSC0003DMT400045488	9.18	81654.03	2232	743	I	√
StLRR-RLK123	PGSC0003DMT400035108	8.7	36677.57	987	328	XI	
StLRR-RLK124	PGSC0003DMT400035086	6.12	66659.15	1803	600	XIII	√
StLRR-RLK125	PGSC0003DMT400027924	6.14	97961.1	2628	875	XIV	
StLRR-RLK126	PGSC0003DMT400038616	6.77	73649.48	2049	682	VI	
StLRR-RLK127	PGSC0003DMT400008475	8.24	68289.56	1848	615	VI	√
StLRR-RLK128	PGSC0003DMT400035585	7.04	74694.66	2016	671	VI	√
StLRR-RLK129	PGSC0003DMT400018675	6.12	112744.05	3057	1018	IX	√
StLRR-RLK130	PGSC0003DMT400069725	7.8	89718.46	2475	824	VI	
StLRR-RLK131	PGSC0003DMT400059959	6.61	68977.84	1851	616	XIII	√
StLRR-RLK132	PGSC0003DMT400010659	6.1	110043.91	3003	1000	XI	
StLRR-RLK133	PGSC0003DMT400053742	5.74	119331.88	3249	1082	XI	√
StLRR-RLK134	PGSC0003DMT400053703	5.94	64866.59	1773	590	XI	

StLRR-RLK135	PGSC0003DMT400053696	7.52	108781.19	2979	992	XI	√
StLRR-RLK136	PGSC0003DMT400053693	5.92	222859.21	6024	2007	XI	√
StLRR-RLK137	PGSC0003DMT400083940	6.36	95462.67	2589	862	IX	
StLRR-RLK138	PGSC0003DMT400077223	5.62	72589.85	1962	653	I	√
StLRR-RLK139	PGSC0003DMT400061545	7.58	92244.61	2574	857	VI	
StLRR-RLK140	PGSC0003DMT400042952	5.76	96557.87	2685	894	VI	√
StLRR-RLK141	PGSC0003DMT400070944	6.4	104693.97	2910	969	VI	√
StLRR-RLK142	PGSC0003DMT400070937	8.49	65611.99	1779	592	VI	√
StLRR-RLK143	PGSC0003DMT400067070	6.03	107734.48	2934	977	IX	√
StLRR-RLK144	PGSC0003DMT400012557	5.62	74959.41	2079	692	IV	√
StLRR-RLK145	PGSC0003DMT400074103	7.59	70824.05	1947	648	VI	√
StLRR-RLK146	PGSC0003DMT400083089	6.36	121763.77	3321	1106	XII	√
StLRR-RLK147	PGSC0003DMT400069633	9.28	70263.69	1878	625	IX	√
StLRR-RLK148	PGSC0003DMT400015087	5.38	107603.24	2937	978	XII	
StLRR-RLK149	PGSC0003DMT400078229	6.06	136161.1	3699	1232	XI	√
StLRR-RLK150	PGSC0003DMT400078298	9.14	69145.9	1890	629	VI	√
StLRR-RLK151	PGSC0003DMT400071183	8.39	67783.22	1842	613	VIII	√
StLRR-RLK152	PGSC0003DMT400030886	6.14	105390.09	2880	959	II	√
StLRR-RLK153	PGSC0003DMT400090109	6.26	94503.8	2568	855	V	√
StLRR-RLK154	PGSC0003DMT400029141	6.28	103165.21	2823	940	XI	
StLRR-RLK155	PGSC0003DMT400028991	6.66	69706.84	1896	631	XIII	√
StLRR-RLK156	PGSC0003DMT400070664	5.55	41345.9	1110	369	VIII	
StLRR-RLK157	PGSC0003DMT400070666	6.83	52325.52	1401	466	VIII	
StLRR-RLK158	PGSC0003DMT400048264	5.47	120673.37	3258	1085	XI	√
StLRR-RLK159	PGSC0003DMT400053338	6.86	130255.9	3543	1180	XI	√
StLRR-RLK160	PGSC0003DMT400054193	5.86	55532.2	1509	502	XI	
StLRR-RLK161	PGSC0003DMT400024732	8.47	72272.81	1944	647	VI	√
StLRR-RLK162	PGSC0003DMT400013987	5.52	111496.31	3075	1024	XI	
StLRR-RLK163	PGSC0003DMT400013991	5.79	83157.02	2253	750	XI	
StLRR-RLK164	PGSC0003DMT400024269	5.6	108655.33	2961	986	IX	√
StLRR-RLK165	PGSC0003DMT400044535	8.4	113290.28	3084	1027	XIII	√
StLRR-RLK166	PGSC0003DMT400079864	6.3	122335.4	3330	1109	IX	√
StLRR-RLK167	PGSC0003DMT400032398	6.19	114990.42	3135	1044	VIII	√
StLRR-RLK168	PGSC0003DMT400057117	6.74	63870.91	1698	565	VI	√
StLRR-RLK169	PGSC0003DMT400057334	5.55	118001.58	3252	1083	IX	√
StLRR-RLK170	PGSC0003DMT400056932	6.08	130150.94	3579	1192	VIII	
StLRR-RLK171	PGSC0003DMT400032577	7.27	129676.28	3534	1177	XI	
StLRR-RLK173	PGSC0003DMT400048435	5.85	108946.29	2973	990	IX	√
StLRR-RLK174	PGSC0003DMT400019757	6.06	114643.67	3099	1032	IX	√
StLRR-RLK175	PGSC0003DMT400077438	7.04	76913	2058	685	IX	√
StLRR-RLK176	PGSC0003DMT400077419	8.77	65553.9	1752	583	IX	
StLRR-RLK177	PGSC0003DMT400077423	8.99	60620.38	1629	542	IX	
StLRR-RLK178	PGSC0003DMT400074940	6.6	105265.52	2841	946	V	√
StLRR-RLK179	PGSC0003DMT400075987	9.12	70064.44	1878	625	VI	

StLRR-RLK180	PGSC0003DMT400019705	6.87	89288.7	2424	807	Ⅺ	
StLRR-RLK182	PGSC0003DMT400050989	6.13	112689.73	3048	1015	Ⅺ	√
StLRR-RLK184	PGSC0003DMT400056576	6.49	109851.66	2949	982	Ⅺ	
StLRR-RLK185	PGSC0003DMT400056585	6.98	120063.42	3258	1085	Ⅺ	√
StLRR-RLK186	PGSC0003DMT400010103	5.88	75043.22	2058	685	Ⅲ	√
StLRR-RLK187	PGSC0003DMT400010074	5.36	95830.85	2649	882	Ⅶ	√
StLRR-RLK188	PGSC0003DMT400031856	5.94	72397.38	1977	658	Ⅰ	√
StLRR-RLK189	PGSC0003DMT400032061	6.56	70855.91	1929	642	Ⅵ	√
StLRR-RLK190	PGSC0003DMT400004363	5.76	102673.38	2787	928	ⅪV	√
StLRR-RLK191	PGSC0003DMT400034324	8.12	93585.44	2601	866	Ⅵ	
StLRR-RLK192	PGSC0003DMT400068892	5.39	102079.65	2817	938	Ⅶ	
StLRR-RLK193	PGSC0003DMT400022521	6.64	102359.65	2829	942	Ⅻ	√
StLRR-RLK194	PGSC0003DMT400045511	5.78	101134.11	2715	904	ⅪV	√
StLRR-RLK195	PGSC0003DMT400040874	6.21	72345.18	1992	663	Ⅵ	√
StLRR-RLK196	PGSC0003DMT400040886	5.14	122834.71	3390	1129	Ⅸ	√
StLRR-RLK197	PGSC0003DMT400022119	5.89	112453.2	3087	1028	Ⅸ	√
StLRR-RLK198	PGSC0003DMT400009696	5.18	133957.99	3660	1219	Ⅸ	√
StLRR-RLK199	PGSC0003DMT400030278	5.46	121917.98	3327	1108	Ⅸ	√
StLRR-RLK200	PGSC0003DMT400076250	5.65	133294.02	3693	1230	Ⅸ	√
StLRR-RLK201	PGSC0003DMT400053083	5.78	101356.77	2799	932	Ⅻ	√
StLRR-RLK202	PGSC0003DMT400055650	6.44	107429.1	2955	984	Ⅶ	
StLRR-RLK203	PGSC0003DMT400055685	5.2	134398.33	3711	1236	Ⅷ	
StLRR-RLK204	PGSC0003DMT400049957	6.69	86202.71	2316	771	Ⅺ	√
StLRR-RLK205	PGSC0003DMT400016636	6.7	112538.75	3030	1009	Ⅺ	√
StLRR-RLK206	PGSC0003DMT400045607	5.69	58697.8	1578	525	ⅫI	
StLRR-RLK207	PGSC0003DMT400084287	8.87	61594.92	1686	561	Ⅱ	√
StLRR-RLK208	PGSC0003DMT400071263	6.49	89986.23	2427	808	ⅪV	
StLRR-RLK209	PGSC0003DMT400080229	8.75	57396.72	1557	518	ⅪV	
StLRR-RLK210	PGSC0003DMT400021662	5.93	105974.27	2895	964	ⅪV	√
StLRR-RLK211	PGSC0003DMT400072330	5.49	102271.01	2805	934	Ⅻ	√
StLRR-RLK212	PGSC0003DMT400072512	8.45	87012.58	2358	785	Ⅰ	√
StLRR-RLK213	PGSC0003DMT400021049	5.69	97775.52	2667	888	Ⅸ	√
StLRR-RLK214	PGSC0003DMT400040459	6.54	69643.63	1944	647	Ⅵ	√
StLRR-RLK215	PGSC0003DMT400041690	5.88	67557.22	1815	604	ⅫI	√
StLRR-RLK216	PGSC0003DMT400002598	7.17	109987.58	3009	1002	Ⅺ	√
StLRR-RLK217	PGSC0003DMT400073837	5.73	101986.97	2817	938	Ⅻ	√
StLRR-RLK218	PGSC0003DMT400015538	6.57	124691.03	3393	1130	Ⅺ	√
StLRR-RLK219	PGSC0003DMT400023894	8.21	99645.33	2760	919	Ⅸ	
StLRR-RLK220	PGSC0003DMT400088620	7.6	33808.8	912	303	Ⅺ	
StLRR-RLK221	PGSC0003DMT400059857	6.46	103186.35	2814	937	Ⅺ	
StLRR-RLK222	PGSC0003DMT400059855	6.02	105512.21	2874	957	Ⅸ	√
StLRR-RLK223	PGSC0003DMT400050673	9.3	71927.95	1911	636	Ⅵ	√
StLRR-RLK224	PGSC0003DMT400007518	8.8	70212.2	1908	635	Ⅵ	√
StLRR-RLK225	PGSC0003DMT400000956	6.91	97414.65	2658	885	Ⅸ	

StLRR-RLK226	PGSC0003DMT400073675	6.06	102805.82	2775	924	XIII	
StLRR-RLK228	PGSC0003DMT400074229	5.99	76594.76	2121	706	IV	√
StLRR-RLK229	PGSC0003DMT400012752	7.03	70643.21	1914	637	I	
StLRR-RLK230	PGSC0003DMT400059664	5.68	117817.32	3237	1078	IX	√
StLRR-RLK231	PGSC0003DMT400039968	6.34	98279.63	2667	888	II	√
StLRR-RLK232	PGSC0003DMT400075495	5.6	66379.23	1767	588	VIII	√
StLRR-RLK233	PGSC0003DMT400075494	5.62	66119.04	1749	582	VIII	√
StLRR-RLK234	PGSC0003DMT400043459	5.52	120560.59	3294	1097	IX	√
StLRR-RLK235	PGSC0003DMT400011891	6.14	100737.32	2766	921	XII	√
StLRR-RLK236	PGSC0003DMT400046218	6.94	70944.15	1959	652	VI	√
StLRR-RLK237	PGSC0003DMT400036257	7.21	47150.53	1290	429	XI	
StLRR-RLK238	PGSC0003DMT400036250	5.5	81576.79	2229	742	XI	
StLRR-RLK239	PGSC0003DMT400036243	6.58	57437.31	1551	516	XI	
StLRR-RLK240	PGSC0003DMT400049019	6.46	52661.88	1422	473	XI	
StLRR-RLK241	PGSC0003DMT400039007	7.04	47894.49	1284	427	XI	
StLRR-RLK242	PGSC0003DMT400077881	6.72	48251.92	1302	433	XI	
StLRR-RLK243	PGSC0003DMT400008826	5.91	66849.19	1815	604	VI	√
StLRR-RLK244	PGSC0003DMT400046446	8.83	48270.62	1311	436	XI	
StLRR-RLK245	PGSC0003DMT400097140	8.34	41708.37	1119	372	XI	
StLRR-RLK246	PGSC0003DMT400025493	8.43	73648.53	1998	665	VI	√
