# Supplementary material for: Identification and Characterization of LRR-RLK Family Genes in Potato Reveal Their Involvement in Peptide Signaling of Cell Fate Decisions and Biotic/Abiotic Stress Responses
Source: Cells. 2018 Aug 27;7(9):120. doi: 10.3390/cells7090120 (PMC6162732; doi:10.3390/cells7090120)
Supplement: Supplementary file 1 [file cells-07-00120-s001.zip › Sup File 4.pdf]

Sup File 4. LRR motifs analysis in the LRR domain of identified potato LRR-RLK members

**LRR**	**1**	**2**	**3**	**4**	**5**	**6**	**7**	**8**	**9**	**10**	**11**	**12**	**13**	**14**	**15**	**16**	**17**	**18**	**19**	**20**	**21**	**22**	**23**	**24**
M02	<u>**G**</u>	x	**I**	**P**/s	x	e	I/l	G	n	**L**	x	x	<u>**L**</u>	x	x	**L**	d	<u>**L**</u>	S	x	<u>**N**</u>	n	**L**/f	T/s
M03	<u>**G**</u>	x	**I**	<u>**P**</u>	x	x	I/L	g	x	L/c	x	x	<u>**L**</u>	x	x	<u>**L**</u>	x	**L**	x	N	<u>**N**</u>	x	**L**/f	s/t
M04	<u>**G**</u>	x	**I**	<u>**P**</u>	x	x	i/l	x	n	L	x	x	<u>**L**</u>	x	x	<u>**L**</u>	n	<u>**L**</u>	q/s	n	<u>**N**</u>	x	**L**/f	S/t
M06	<u>**G**</u>	x	I/l	**P**	x	e	l/i	G	x	l	x	s	<u>**L**</u>	x	x	<u>**L**</u>	D	<u>**L**</u>	S	x	<u>**N**</u>	x	L/F	T/s
M07	<u>**G**</u>	x	**I**	<u>**P**</u>	x	s	L/f	G	x	**L**	x	x	<u>**L**</u>	x	x	<u>**L**</u>	x	<u>**L**</u>	s	x	<u>**N**</u>	x	**L**	S
M08	<u>**G**</u>	x	**I**	**P**	x	E	I/L	G	x	L	x	x	<u>**L**</u>	x	x	<u>**L**</u>	x	<u>**L**</u>	x	x	<u>**N**</u>	x	**L**/f	T
M09	<u>**G**</u>	x	**I**	<u>**P**</u>	x	x	I/l	g	x	L	x	x	<u>**L**</u>	x	x	<u>**L**</u>	x	**L**	x	x	<u>**N**</u>	x	**L**/f	S
M10	**G**	x	**I**	<u>**P**</u>	x	s	I	G	n	**L**	x	x	<u>**L**</u>	e	x	**L**	n/d	L	**S**	x	<u>**N**</u>	x	**L**/F	s
M11	<u>**G**</u>	x	I	<u>**P**</u>	x	s	L/I	G	n	**C**	x	S	<u>**L**</u>	x	x	**L**	x	<u>**L**</u>	s	x	<u>**N**</u>	x	**L**	S
M12	**G**	x	I	P	x	e	I/f	**G**	x	<u>**L**</u>	x	x	<u>**L**</u>	x	v	<u>**L**</u>	d	<u>**L**</u>	x	x	<u>**N**</u>	n	L/f	S
M13	<u>**G**</u>	x	**I**	<u>**P**</u>	x	e	l/i	**G**	k	l/f	x	x	<u>**L**</u>	x	x	<u>**L**</u>	d	**L**	<u>**S**</u>	x	<u>**N**</u>	x	<u>**L**</u>	T
M14	<u>**G**</u>	x	I/l	**P**	x	x	i/l	G	x	**L**	x	x	<u>**L**</u>	x	x	**L**	**D**	L	**S**	x	<u>**N**</u>	x	L/F	S/t
M15	<u>**G**</u>	x	**I**	<u>**P**</u>	x	s	L/I	**G**	x	**L**	x	x	<u>**L**</u>	x	x	<u>**L**</u>	x	**L**	x	x	<u>**N**</u>	x	L/F	S
M16	<u>**G**</u>	x	I/l	<u>**P**</u>	p	x	**L**	**C**	s	g	x	k	<u>**L**</u>	x	x	**L**	i	**L**	x	x	<u>**N**</u>	x	**L**/F	t
M23	<u>**G**</u>	x	I/l	**P**	x	x	L	g	x	L	x	x	<u>**L**</u>	x	x	**L**	x	**L**	x	x	<u>**N**</u>	x	L/F	s/t
M24	<u>**G**</u>	x	I	<u>**P**</u>	x	x	L	g	x	**L**	x	x	<u>**L**</u>	x	x	**L**	D/n	L	S	x	<u>**N**</u>	x	L/F	t
M25	<u>**G**</u>	x	**I**	<u>**P**</u>	x	x	I	**G**	n	l	x	x	**L**	x	x	**L**	**D**	L	**S**	x	<u>**N**</u>	x	**L**/f	s
M26	<u>**G**</u>	x	I/l	**P**	x	x	L	g	n	**L**	t/s	x	**L**	x	x	L	d	L	s	n	<u>**N**</u>	n	L/f	S
M27	<u>**G**</u>	x	**I**/l	<u>**P**</u>	x	e	L/i	G	n	l	x	n	**L**	x	x	<u>**L**</u>	x	**L**	x	x	<u>**N**</u>	x	**L**/f	s
M28	<u>**G**</u>	x	**I**	<u>**P**</u>	x	x	I/l	G	n	l	x	x	<u>**L**</u>	x	x	l	d	L	s	x	<u>**N**</u>	x	L/F	S/t
M29	<u>**G**</u>	x	I/L	<u>**P**</u>	x	E	l	G	N	C	x	s	<u>**L**</u>	x	x	L	d/n	L	S	x	<u>**N**</u>	x	L/F	s
M30	G	x	I	**P**	P	e	I/l	g	x	**L**	x	n	**L**	x	x	L	x	L	x	x	<u>**N**</u>	n	F/L	T
M32	<u>**G**</u>	x	**I**/l	**P**	x	x	i/l	x	x	L	s	x	<u>**L**</u>	x	x	L	**D**	**L**	**S**	x	<u>**N**</u>	x	L/f	x
M33	<u>**G**</u>	s	I/L	**P**/s	x	x	l/i	x	x	L	x	s	<u>**L**</u>	x	x	L	x	L	x	n	<u>**N**</u>	x	L/F	S
M34	<u>**G**</u>	x	I	<u>**P**</u>	x	e	L/I	g	x	C	x	x	<u>**L**</u>	x	x	**L**	d	**L**	S	x	<u>**N**</u>	x	L	s
M35	<u>**G**</u>	x	**I**	<u>**P**</u>	x	e	I	**G**	N	L	x	x	<u>**L**</u>	x	x	**L**	x	L	s	x	<u>**N**</u>	x	L/F	S
M36	<u>**G**</u>	x	<u>**I**</u>	<u>**P**</u>	x	x	l/f	g	n	x	x	x	<u>**L**</u>	x	x	L/f	D/n	L	S	x	<u>**N**</u>	x	L/f	S
M37	<u>**G**</u>	e	i/l	**P**	x	e	i/l	G	x	L	x	x	<u>**L**</u>	x	x	L	d	L	x	x	<u>**N**</u>	x	**F**/l	S
M38	<u>**G**</u>	x	**I**	<u>**P**</u>	x	e	L/i	G	x	L	x	x	<u>**L**</u>	x	x	<u>**L**</u>	d	**L**	s	x	<u>**N**</u>	x	**L**	S/t

Note: The sequence conservation of LRR motif at each position was measured by bits. If the bits value of the amino acid at certain position is less than 0.5, such position would be represented with x; 1 > bits ≥ 0.5, with lowercase; 2 > bits ≥ 1, with capital letter; 3 > bits ≥ 2, with bold capital; bits ≥ 3, with underlined capital letter in bold.
