# Supplementary figures and images for: Identification and Characterization of LRR-RLK Family Genes in Potato Reveal Their Involvement in Peptide Signaling of Cell Fate Decisions and Biotic/Abiotic Stress Responses
Source: Cells. 2018 Aug 27;7(9):120. doi: 10.3390/cells7090120 (PMC6162732; doi:10.3390/cells7090120)

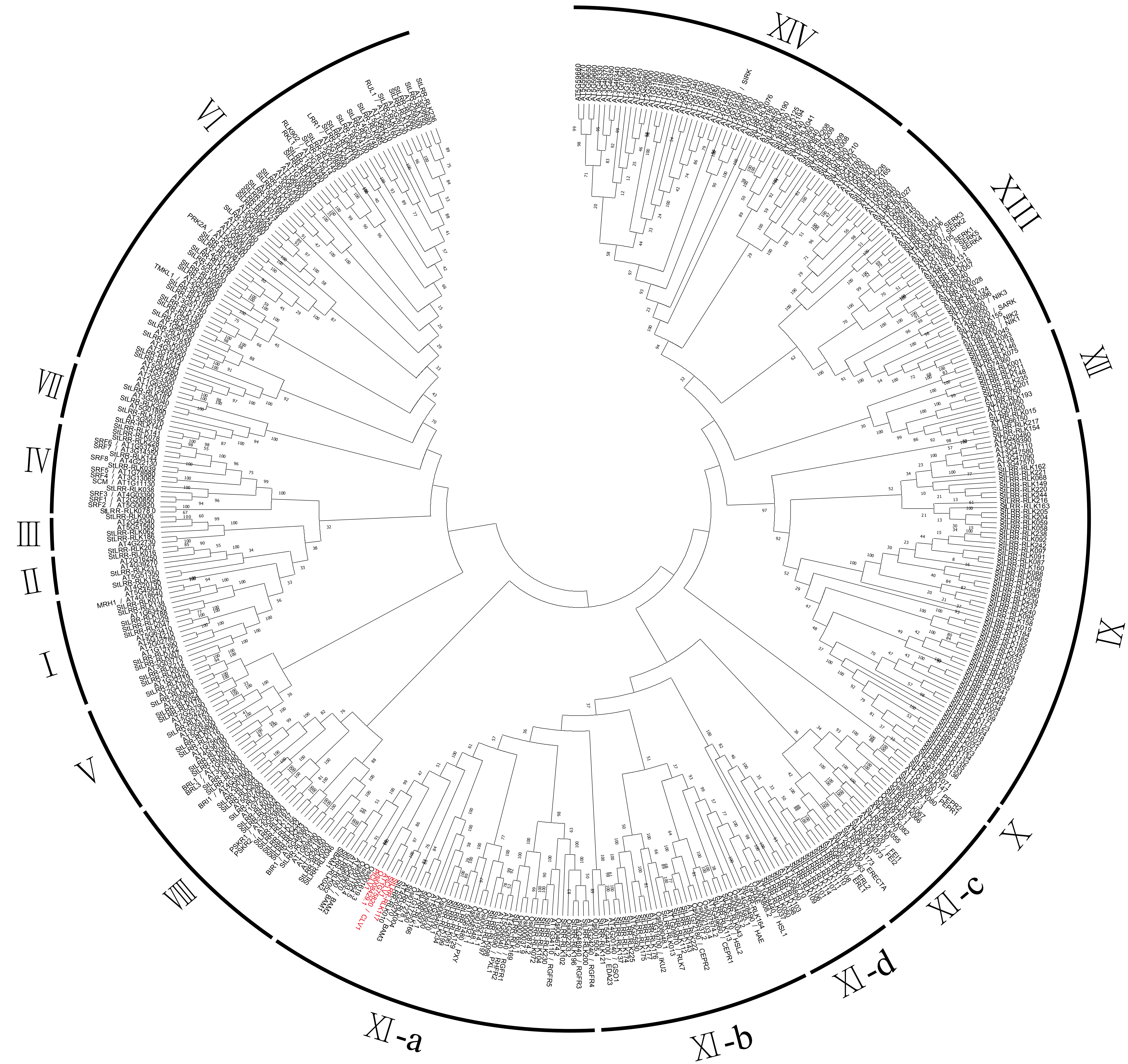

Supplement: Supplementary file 1 [file cells-07-00120-s001.zip › Sup File 3.pdf]

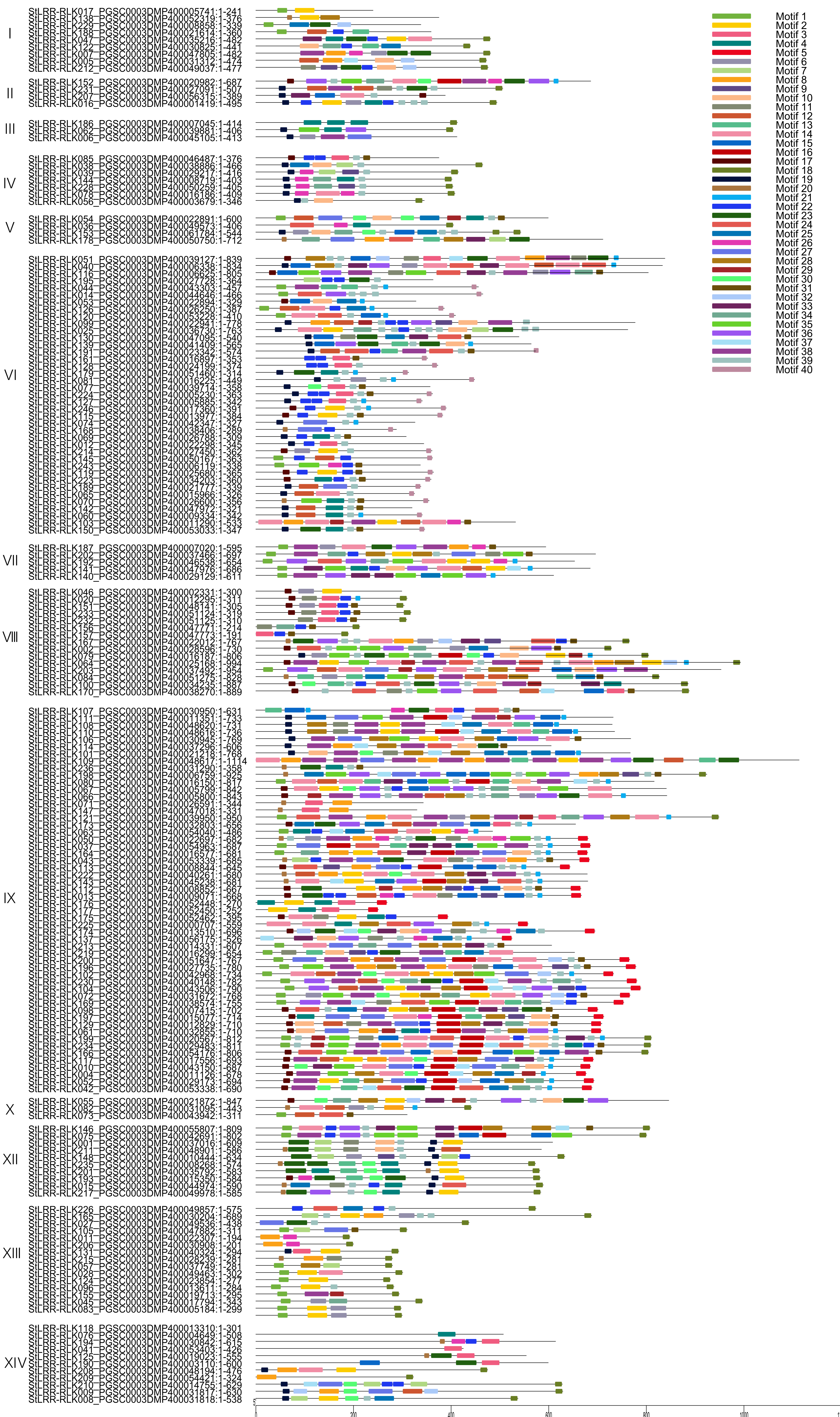

Supplement: Supplementary file 1 [file cells-07-00120-s001.zip › Sup File 5.pdf]

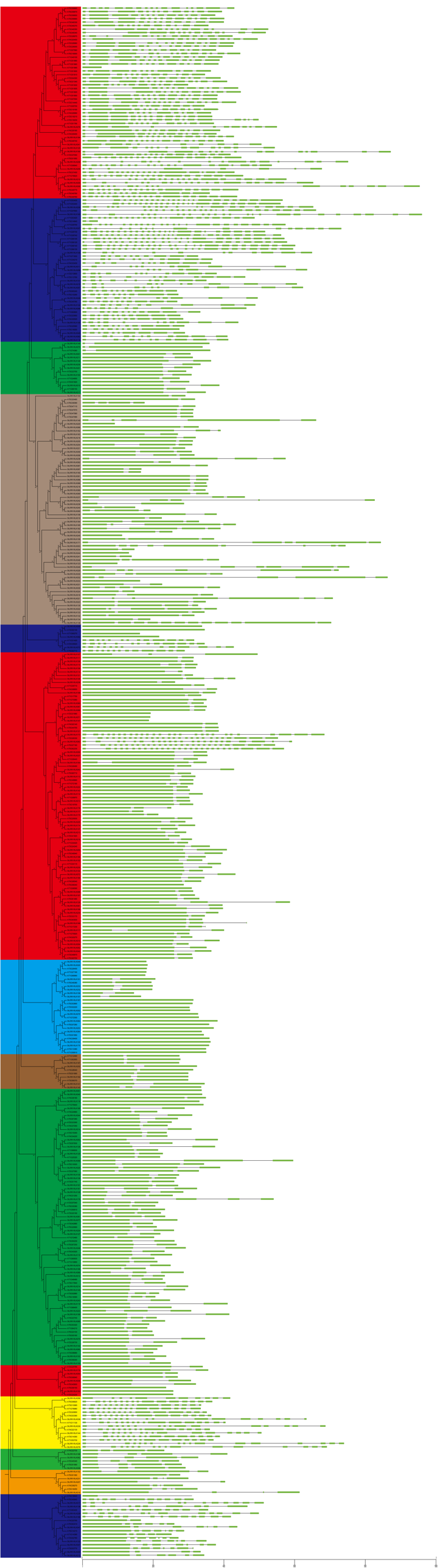

Supplement: Supplementary file 1 [file cells-07-00120-s001.zip › Sup File 6.pdf]
